# SWEET Transporters for the Nourishment of Embryonic Tissues during Maize Germination

**DOI:** 10.3390/genes10100780

**Published:** 2019-10-07

**Authors:** Montserrat López-Coria, Tomás Sánchez-Sánchez, Víctor Hugo Martínez-Marcelo, G. Paulina Aguilera-Alvarado, Mireya Flores-Barrera, Beatriz King-Díaz, Sobeida Sánchez-Nieto

**Affiliations:** Departamento de Bioquímica, Facultad de Química, UNAM, Ciudad de México 04510, Mexico; lopez_coria@comunidad.unam.mx (M.L.-C.); tomassanchez328@gmail.com (T.S.-S.); victor.hugo.martinez.vhm@gmail.com (V.H.M.-M.); cirapaulalocaaa@hotmail.com (G.P.A.-A.); maraya_360@hotmail.com (M.F.-B.); kingbeat@unam.mx (B.K.-D.)

**Keywords:** SWEET transporters, ZmSWEET, germination, maize embryo, diffusional transport

## Abstract

In maize seed germination, the endosperm and the scutellum nourish the embryo axis. Here, we examined the mRNA relative amount of the SWEET protein family, which could be involved in sugar transport during germination since high [^14^-C]-glucose and mainly [^14^-C]-sucrose diffusional uptake were found in embryo tissues. We identified high levels of transcripts for SWEETs in the three phases of the germination process: ZmSWEET4c, ZmSWEET6b, ZmSWEET11, ZmSWEET13a, ZmSWEET13b, ZmSWEET14b and ZmSWEET15a, except at 0 h of imbibition where the abundance of each ZmSWEET was low. Despite the major sucrose (Suc) biosynthesis capacity of the scutellum and the high level of transcripts of the Suc symporter SUT1, Suc was not found to be accumulated; furthermore, in the embryo axis, Suc did not decrease but hexoses increased, suggesting an efficient Suc efflux from the scutellum to nourish the embryo axis. The influx of Glc into the scutellum could be mediated by SWEET4c to take up the large amount of transported sugars due to the late hydrolysis of starch. In addition, sugars regulated the mRNA amount of SWEETs at the embryo axis. These results suggest an important role for SWEETs in transporting Suc and hexoses between the scutellum and the embryo axis, and differences in SWEET transcripts between both tissues might occur because of the different sugar requirements and metabolism.

## 1. Introduction

Sucrose (Suc) is the main carbohydrate synthesized during photosynthesis, and is mainly exported through the vascular system and distributed to non-photosynthetic sink tissues to support its growth and development. Apoplastic transport depends on several families of transporters: SUTs (SUcrose Transporter) and STPs (Sugar Transporter Protein) are families of Suc and hexose transporters, respectively, that drive the accumulation of sugars into the cells or vacuoles at the expense of the proton gradient [1,2]. The SWEET family is composed of transporters that move Suc or hexoses in the direction of the concentration gradient at the plasma membrane or tonoplast [3]. SWEETs are implicated in multiple physiological processes that require a large movement of sugars. For instance, Suc efflux from the parenchyma phloem cells is mediated by SWEET11 and 12 in *Arabidopsis thaliana* [3,4]. Meanwhile, in *Zea mays*, SWEET13a, b and c are essential for allocating the sugars from the photosynthetic cells to the phloem [5]. Sugar secretion from the nectar parenchyma in eudicots is produced by AtSWEET9 [6]. OsSWEET11 is required to supply sugars during pollen development, and its suppression induces male sterility in rice [7]. VuSWEET10 mediates sugar accumulation in grapes [8]. AtSWEET11, 12 and 15 are necessary for embryo development, and their absence reduces the starch and lipid content and seed weight [9]. SWEET4c contributes hexoses during the endosperm filling in maize and rice [10].

Seed germination is a process that begins with water uptake; the hydration initiates a cascade of metabolic activities and mobilizes the spatial and temporal use of the storage reserves to support the radicle protrusion by the embryo axis and, subsequently, the seedling growth [11]. The transporters that are involved in such massive metabolic mobilization have not been fully identified. In maize, two tissues sustain the embryo axis development, the scutellum and the endosperm. The scutellum is close to the embryo axis and is rich in lipids and capable of Suc synthesis [12]. The endosperm surrounds the scutellum and consists mainly of dead cells full of starch and other nutrients; its external surface is made of a single layer of living cells called aleurone. The scutellum and the aleurone layer provide the endosperm sugar, protein and lipid hydrolyzing enzymes near the time of the radicle protrusion [12,13]. The endosperm is separated from the scutellum by a fibrous layer made of phenols, neutral lipids and β-glucans [13,14]; this separation may explain why the scutellum contains membrane transporters such as the plasma membrane H^+^-ATPase and the SUTs [15,16]. Transcripts of several members of SUTs were found in the scutellum: TaSUT1 at the epidermis, ZmSUT1 in the parenchyma cells, and SUTs from rice, castor bean and wheat in the companion cells of the fully developed vascular system [17,18,19,20], which is embedded in the parenchyma cells [20]. The scutellum is attached to the embryo axis at the scutellar node, in which the vascular systems of the root and the embryonic leaves converge [20]. Cell domains connected by plasmodesmata are part of the vascular systems of the scutella and embryo axis, creating a symplastic pathway where the nutrients could be distributed [14,15,21]. However, the mobilization of sugars from the storage tissues to the embryo axis could also occur by diffusional transporters such as the SWEETs, since in mature leaves symplastic and apoplastic pathways allow the mobilization of Suc to the phloem [3]. In maize, SWEET4c is important for the provision of Glc to the starch biosynthesis in the endosperm during the embryogenesis [10], but its contribution to the germination process is unknown. ZmSWEET4c may contribute to the sugar transport during germination along with the other SWEETs. Therefore, the main goal of this work was to identify the maize SWEETs that might contribute to the nourishment of the embryo axis during the seed germination process.

As described before, the embryo axis germinates while surrounded by the scutellum and endosperm; both tissues provide the nutrients necessary to sustain the embryo axis’ growth. However, the embryo axis can germinate without the endosperm [16], suggesting that, in the scutellum and embryo axis, there are enough nutrients and metabolic and transport capacity to ensure the germination. Therefore, in the two main tissues that comprise the embryo—the scutellum and embryo axis—we analyzed the sugar and lipid content, the sugar transport activity and the relative messenger RNA (mRNA) levels of several genes, including the SWEETs during the germination process.

## 2. Materials and Methods 

### 2.1. In Silico Analysis of the SWEET Family in Maize

Gene sequences of *Z. mays* SWEETs were obtained from the Plaza 4.0 comparative genomic platform [21] and the Aramemnon database [22]. To identify the conserved motifs in the SWEET protein sequences, the Pfam 31.0 database was used [23], and for analysis of the transmembrane helices, the TMHMM Server version 2.0 was utilized [24].

### 2.2. Plant Material

Maize seeds VS535 treated with the deltametrine insecticide and the interthiam fungicide by a commercial supplier were washed and disinfected with 2% household bleach for 2 min and rinsed with sterile deionized water at least 3 times. Seeds were incubated at 29 °C under darkness in plates with 1% agar alone or supplemented with 5 or 50 mM of Suc, Glc and Mannitol (used as an osmotic control). After incubation, the embryo, embryo axis and scutellum were isolated manually using a razor blade as reported by [16,17]. The tissue from 0 h of germination was extracted from dry seeds.

### 2.3. Determination of the Phases of Germination by Evaluating the Water and Oxygen Uptake

Seeds were incubated in 1% agar at 0, 5, 10, 15, 20, 25 and 30 h, then embryos were excised by hand and weighed to obtain the fresh weight. To record the oxygen uptake, 10 embryos of each imbibition time were immersed in 10 mL of distilled water with constant agitation. Oxygen uptake was monitored for 4–5 min with a YSI 5300 oximeter (Yellow Springs Instrument Co., Inc. Yellow Springs, OH, USA) using a Clark Electrode. Curves obtained were used to determine the 3 phases of germination for the maize seeds VS535. After 30 h the percentage of germinated seeds was 100 ± 5.

### 2.4. RNA Extraction

RNA from embryos and embryos axis isolated from maize seeds imbibed by 0, 18, 30 and 48 h were obtained using TRIzol (Invitrogen, Carlsbad, CA, USA) according to the manufacturer’s instructions. Total RNA was quantified on a NANODROP 2000 (Thermo Scientific Inc. Waltham, MA, USA). RNA samples with ratios of absorbance at 260 nm and 280 nm close to 2.0 were used in the next experiments [25]. The integrity of the RNA was evaluated on a 2% agarose gel (Figure S1). The RNA (1 µg) was mixed with 1 µL oligo dT 20 µM and nuclease-free water to complete 10 µL, denatured at 70 °C for 15 min and chilled in ice for 5 min. The ImProm-II™ Reverse Transcription System from Promega (Madison, WI, USA) was used to obtain the cDNA, and the reaction conditions included 1 cycle of annealing at 25 °C for 5 min, extension at 42 °C for 60 min, and denaturing at 70 °C for 15 min for only 1 cycle. The cDNA was stored at 20 °C until use. The integrity and purity of the RNA was confirmed (Figure S1).

### 2.5. Determination of ZmSWEET and α-Amylase mRNA Levels by RT-PCR

The RT-PCR analysis of the *ZmSWEETs*, α-amylase and Zm18 from 3 independent biological samples was performed using PCR Master Mix according to the manufacturer’s instructions (Promega, Madison, WI, USA). In brief 2 µL cDNA, 0.5 µL forward and reverse primers 20 µM, 12.5 µL PCR Master Mix and 9.5 µL nuclease-free water were mixed in ice. The RT-PCR reaction conditions were pre-denaturation at 94 °C for 5 min, 35 cycles of denaturation at 94 °C for 40 s, annealing at 57 °C for 1 min, extension at 72 °C for 40 s, and final extension at 72 °C for 7 min. The PCR products were resolved in 1.5% agarose gel. Densitometry analysis was undertaken using the ImageJ program [26]. The primers used for the PCR reaction are in Table S1. All the primers were designed using cDNA sequences of *Zea mays* B73_Ref_Gen_v4 of the EnsemblPlants database for each gene [27], and using the Primer3Plus web interface [28] to amplify an exon region of the SWEET as shown in Figure S2. The Basic Local Alignment Search Tool (BLAST) and alignment analysis tools of the NCBI database Nucleotid-BLAST and PRIMER-BLAST were used to determine the specificity of each pair of primers [29]. None of the primers form homodimers, heterodimers and secondary structures, according to the analysis run using the OligoAnalyzer Tool from the Integrated DNA Technologies (IDT) web page. 

### 2.6. Analysis by qPCR of mRNA of ZmSWEET, Malate Synthase, Sucrose Phosphate Synthase, and Sucrose Transporter 1 in Scutellum and Embryo Axis

Quantitative PCR was conducted to analyze the mRNA amount of the ZmSWEETs, MAS, SPS and SUT1. The efficiency was calculated using a calibration curve with a serial dilution of cDNA and the following formula: E = (10[−1/m]) * 100, where m is the slope curve of Ct vs. the logarithmic DNA concentration (Tables S2 and S3). To ensure the reliability of the results, the qPCR specificity and efficiency of each pair of primers used to amplify the SWEETs were analyzed (Figure S3) and no-RT control (RNA instead of cDNA) was added (Table S4). The qPCR was performed in a 7500 Real-Time PCR System (Applied Biosystems a division from Thermo Scientific Inc. Waltham, MA, USA). The PCR reaction mix contained 10 µL SYBR Green Master Mix (Applied Biosystems), 0.15 μL forward primers 20 µM, 0.15 μL reverse primers 20 µM, 2 μL cDNA, and 7.7 μL nuclease-free water. The reaction conditions included a holding state at 95 °C for 10 min; a cycling stage of 40 cycles at 95 °C for 15 s and 60 °C for 1 min; and a melting curve stage at 95 °C for 15 s and 60 °C 1 min. The mRNA amount of Zm18s [9] was used for the control. The ratio of transcripts was calculated using the following formula [30]:

Ratio transcripts = E_target_^(ΔCPtarget(control-sample)^/E_ref_^(ΔCPref(control-sample)^ (1)

where E_target_ is the amplification efficiency of the target gene, ΔCP_target_ is the C_t_ value of the control sample minus the C_t_ value of the treated samples, E_ref_ is the amplification efficiency of the housekeeping gene, and ΔCP_ref_ is the C_t_ value of the housekeeping gene in the control sample minus the C_t_ value of the housekeeping gene in the treatment samples [30].

### 2.7. Determination of Soluble Sugars, Starch and Lipid Content

The soluble sugars were extracted by grinding 200 mg of scutellum or embryo axis with 5 mL of 80 ethanol at 80 °C. The aqueous phase was centrifuged 13,000× *g* for 10 min. The supernatants of the two extracts were combined, heated to 70 °C and evaporated to dryness, and used to quantify the soluble sugars. The pellet contained starch. The content of Glc, fructose (Fru) and Suc was determined using an enzymatic assay coupled to the reduced β-nicotinamide adenine dinucleotide (NADH) production using the Glc assay reagent (GAR, Sigma-Aldrich, St. Louis, MO, USA) in a 96-microplate format, according to [16]. In short, to determine the Glc and Fru, a 12 µL sample was mixed with 200 µL GAR; the reaction was incubated for 20 min at 25 °C, and the NADH was detected at 340 nm. To quantify the Fru, 2 µL of 1.2 U/mL phosphoglucose isomerase was added to the previous mixture, incubated for 5 min at 25 °C, and the increase in the NADH was detected at 340 nm with a microplate reader (Thermo Fisher Scientific, Waltham, MA, USA). The Suc content was determined by subjecting the sample to enzymatic hydrolysis under acidic conditions to produce Fru and Glc, using a 12 µL sample and 4 µL of 80 mg/mL invertase (Sigma-Aldrich, St. Louis, MO, USA; dissolved in 200 mM magnesium acetate pH 5.0), followed by 2 h incubation at 37 °C. Then, the released hexoses were quantified as described above. The starch content was estimated first by solubilization with 1 mL of water heated to 90 °C for 4 h, and then was degraded with 1 volume of 14 mg/mL amyloglucosidase in acetate buffer (Roche, Switzerland). The produced Glc was measured with the reaction with GAR, as described above. Lipids were extracted according to [31]. Briefly, 1 g of tissue was mixed with cold CHCl3:MetOH 2:1, and the mixture was filtered by 4 gauze layers and then centrifuged at 3000 rpm for 5 min. The organic phase was loaded into microtubes, and the CHCl_3_ was evaporated to dryness. The lipid content was determined by weight.

### 2.8. Determination of Cell Wall and Vacuolar Invertase Activity

Soluble and cell wall invertase (CWINV) extracts were obtained from the embryo and embryo axis essentially as reported by Pelleschi et al. [32]. The embryo was used instead of the scutellum since the embryo axis has very low INV activity. The CWINV and vacuolar invertase (VINV) activity was determined by an enzymatic assay coupled to the reduction of β-nicotinamide adenine dinucleotide phosphate (NADPH), as described by Bergmeyer and Bemt [33]. The reaction began by adding 200 µg of protein and incubating the reaction for 45 min at 30 °C; at the end the absorbance was measured at 340 nm.

### 2.9. Sugar Uptake in Embryonic Tissues

The sugar uptake of the scutellum and embryo axis was determined according to [16]. Approximately 100 µg of embryo axis or scutellum was imbibed in 500 µL reaction medium containing 50 mM HEPES (4-(2-hydroxyethyl)-1-piperazineethanesulfonic acid) pH 6.0, 300 mM sorbitol, 5 mM KCl, 10 mM Glc or Suc and 1 µCi [^14^-C(U)]-Glc or [^14^-C(U)]-Suc. The tissues were incubated for 15 min at 25 °C, the reaction medium was discarded, and 200 µL of wash medium (50 mM HEPES pH 6, 300 mM sorbitol, 5 mM KCl, 100 mM Glc or Suc) was added and incubated for 5 min, with the buffer being discarded on completion. The tissues were loaded on filter paper for 5 min. The dry tissues were immersed in 500 µL of lysis medium (H_2_O_2_:HCl_4_, 2:1) and incubated overnight. Finally, 300 µL of the solution was loaded in 3 mL scintillation liquid (0.3% 2,5-Diphenyloxazole (PPO), 3.7% Ethylene glycol, 10.6% Ethanol, 25.7% Triton X-100, and 60% Xylol). Determination of the ^14^C-labelled sugars was made in a Beckman Coulter LS 6500 Multi-Purpose Scintillation Counter, with 3 min reading.

The Glc diffusional uptake was determined in the presence of 100 µM carbonyl cyanide m-chlorophenyl hydrazine (CCCP), a protonophore that allows the balance of proton concentration in both sides of the membrane and eliminates the contribution of the Glc active transport. To obtain the Suc diffusional transport, the uptake assays were made in the presence of 2.5 mM p-chloromercuribenzene sulfonate (PCMBS), which is a sulfhydryl group modifier used classically as a sucrose/H^+^ symporter inhibitor [34].

## 3. Results

### 3.1. Maize SWEET Family

According to Eom et al. [35] and Li et al. [36], 24 genes comprise the SWEET family in maize. All putative maize SWEET proteins possess two MtN3/saliva domains, and most are predicted to have the seven transmembrane helices typical of SWEET transporters, except for ZmSWEET4a and ZmSWEET6a, which are predicted to have six helices (Figure S4). Nonetheless, they were considered SWEET family members.

It has been reported that SWEET members are grouped in four clades [35,36,37]. Maize SWEETs are distributed among the four clades, but most fall into clade III, which also groups most of the SWEET transporters from *A. thaliana* and rice. Grouping in a clade does not give information about the physiological function of each SWEET but gives insight into their sugar specificity. Clade I and II have members with Glc transport activity, clade III group members have Suc transport capacity, and some Fru transporters are in clade IV [35,38].

### 3.2. Transcripts for Several SWEETs Were Found at the Embryo Axis and the Scutellum During the Germination

**Figure 1:**
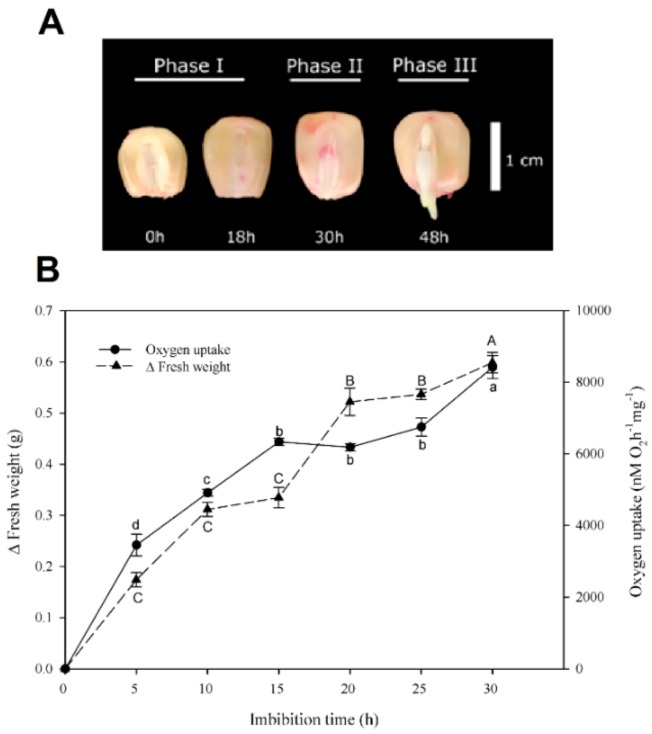
Seeds germinated at different times and embryo hydration and oxygen uptake during germination. (**A**) Seeds were germinated at different times; the germination phases are indicated. (**B**) Hydration and oxygen uptake curves of embryos isolated from imbibed seeds. Seeds were imbibed in agar 1% and incubated at 29 °C at the indicated times; later, the fresh weight and oxygen uptake of isolated embryos were determined. The values are the average of three independent experiments ± SD. Different letters indicate statistically significant values according to the Tukey test, *p* < 0.05. Capital letters indicate oxygen uptake and lowercase letters indicate fresh weight.

To follow the mRNA levels of ZmSWEETs during germination, the three germination phases of the maize seed VS535 were identified. The seeds were germinated in agar (Figure 1A) and the embryos were isolated from them. The first phase of germination was found between 0 and 18 h of imbibition, in which a high increase in embryo fresh weight and oxygen uptake was detected. Phase II starts with the reduction of the water and oxygen uptake and ends with the radicle protrusion at ~30 h of imbibition (Figure 1B). Phase III of germination, also named a post-germinative phase, is also identified as seedling growth and characteristically shows an additional increase in the water and oxygen uptake due to the emergence of the coleoptile (in which five to six primitive leaves are enclosed) and the radicle [39]. The scutellum and embryo axis were isolated from seeds at 0, 18, and 30 h of imbibition to study phases I and II, and at 48 h to include phase III (Figure 1A). 

To determine the presence of SWEETs mRNA in maize embryo tissues, an RT-PCR analysis was undertaken. We were able to amplify specific short sequences for 14 of the 24 *ZmSWEET* genes using RNA from the maize embryos at the different stages of germination (Figure S5). Agarose-gel band intensities were analyzed by densitometry. According to the transcript levels, three groups of SWEETs were identified: *ZmSWEET*s that show an mRNA increment along germination (*ZmSWEET*3b, 4b, 4c, 6b, 11, 12a, 13a, 13b and 14a); *ZmSWEETs* without transcript level changes (*ZmSWEET*1b, 2, 15a, 17); and ZmSWEET mRNAs that are present mostly in the post-germinative phase (*ZmSWEET*14b). Genes of each group were selected to analyze their level of mRNA along the germination process by RT-qPCR.

**Figure 2:**
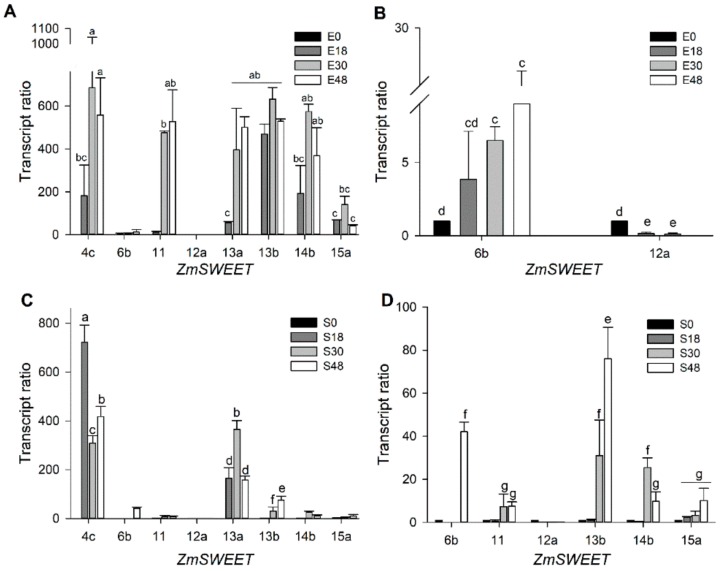
*ZmSWEET* mRNA levels profile in (**A**,**B**) the embryo axis and (**C**,**D**) the scutellum at different hours of germination. To visualize the transcript amount of *SWEETs* that change during germination but that are poorly represented in the tissue, (**B**) and (**D**) show the data at a lower scale than (**A**) and (**C**). The transcript ratio was calculated according to the Pfaffl method [30], normalizing with the invariant housekeeping gene *Zm18s* and the mRNA level of each *ZmSWEET* from tissues that were obtained from non-germinated seeds (0 h). Bars represent the average of two technical replicates from two independent experiments ± SD. Different letters indicate statistically significant values according to the Tukey test, *p* < 0.05.

A low abundance of transcripts for all the analyzed SWEETs was detected at 0 h. A high level of mRNA for *ZmSWEET*4c and 13a was found in both tissues during germination (Figure 2). However, the set of SWEET transcripts that were most abundant differed between the tissues. In the imbibed embryo axis, the most abundant transcripts were ZmSWEET 4c, 11, 13a, 13b, and 14b (Figure 2A,B). In the scutellum, ZmSWEET4c and 13a were highly represented (Figure 2C,D). *ZmSWEET*s detected in the scutellum and embryo axis are included in clades II and III [9,34], which suggests that, at the plasma membrane or tonoplast, the Suc and Glc transport can take place in both tissues.

### 3.3. Metabolic Activity during Germination Reveals High Suc Content in Maize Embryonic Tissues

**Figure 3:**
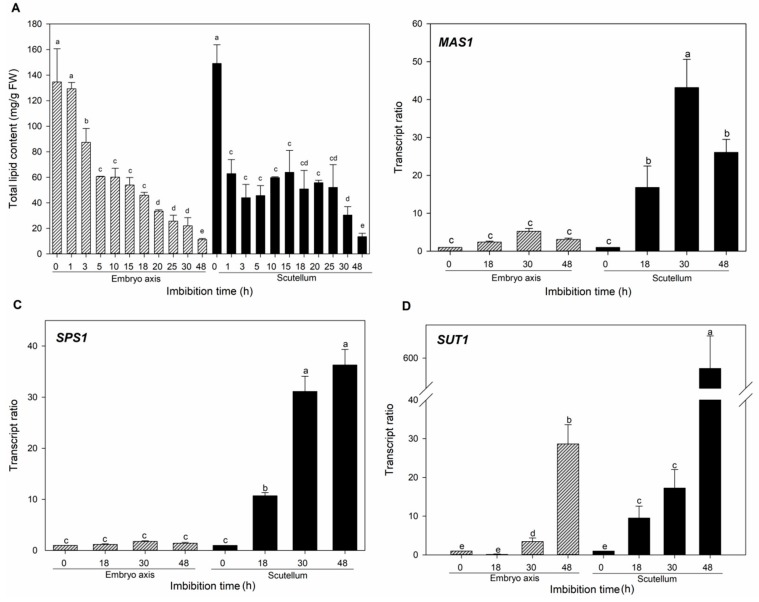
Lipid content (**A**) and RT-qPCR relative transcript amount of MAS (**B**), SPS (**C**), and SUT1 (**D**) in the embryo axis and scutellum at different imbibition times. Bars represent the average of triplicates of two independent experiments ± SD. Ratio transcript was calculated according to the Pfaffl method [30], normalizing with the invariant housekeeping gene *18s* and also with the value of the mRNA amount found in the tissues from non-germinated seeds (0 h). Different letters indicate statistically significant values according to the Tukey test, *p* < 0.05.

Simple and complex molecules are mobilized during germination to sustain the high metabolic activity that allows the embryo axis to develop into a seedling [11]. Variations in lipids, soluble sugars and starch content were compared between the scutellum and embryo axis during germination to understand the possible paths of the soluble sugars. In maize, the lipids, mainly triacylglycerol, accumulate at the scutellum during the embryogenesis [40]. However, as the germination proceeds, major degradation of the lipid occurs at the scutellum, and the lipid content also decreases at the embryo axis (Figure 3A). The lipid mobilization in both tissues could be due to the activation of the glyoxylate cycle to contribute to the Suc biosynthesis. The levels of mRNA of two genes that encodes key enzymes in the Suc synthesis—MAS (glyoxylate cycle enzyme) and SPS (Suc biosynthesis enzyme)—were increased with the imbibition time only at the scutellum (Figure 3B,C), which suggests the active synthesis of Suc in that tissue. In addition, a high number of transcripts of *SUT1*, a Suc symporter, during the 48 h of imbibition was found (Figure 3D). The *MAS*, *SPS* and *SUT1* transcript levels at the scutellum suggests that high Suc accumulation is taking place in that tissue [16,19]. 

**Figure 4:**
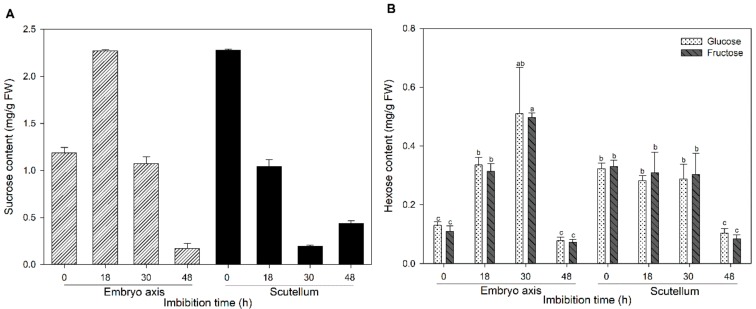
Sucrose (**A**) and hexose (**B**) content in tissues at different imbibition times. Bars show the content of soluble sugars from two independent experiments with triplicates ± SD. Different letters indicate statistically significant values according to the Tukey test, *p* < 0.05.

**Figure 5:**
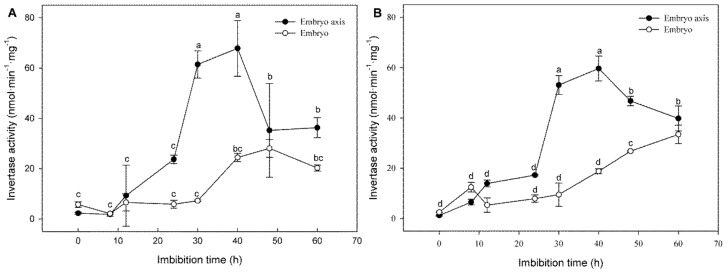
Invertase activity during germination. (**A**) Cell wall invertase, CWINV; (**B**) vacuolar invertase, VINV. The values correspond to the average of two independent experiments with triplicates ± SD. Different letters indicate statistically significant values according to the Tukey test, *p* < 0.05.

However, the Suc content at the scutellum decreases with the imbibition time, in contrast to the steady or high level of Suc in the embryo axis (Figure 4A). In addition, the Suc reduction during germination is not coordinated with an increase in the hexose content at the scutellum, but a high level of hexoses was detected in the embryo axis (Figure 4B). To explore the contribution of the metabolism to the production of the hexoses, the invertase activity was determined. A substantial increase in CWINV and VINV activities in the embryo axis was detected from 0 and 24 h; however, the highest activity was reached later (Figure 5). At the embryo, which contains the scutellum and embryo axis, the CWINV and VINV activities were low before phase III of germination. These results explain the increase in the hexose content in the embryo axis, and the need for the embryonic tissues to transport Suc, since it is possible that the invertases do not use all the Suc available during the first two phases of the germination. However, the embryo axis has low Suc biosynthesis capacity, and therefore, the hexoses may come from other sources.

**Figure 6:**
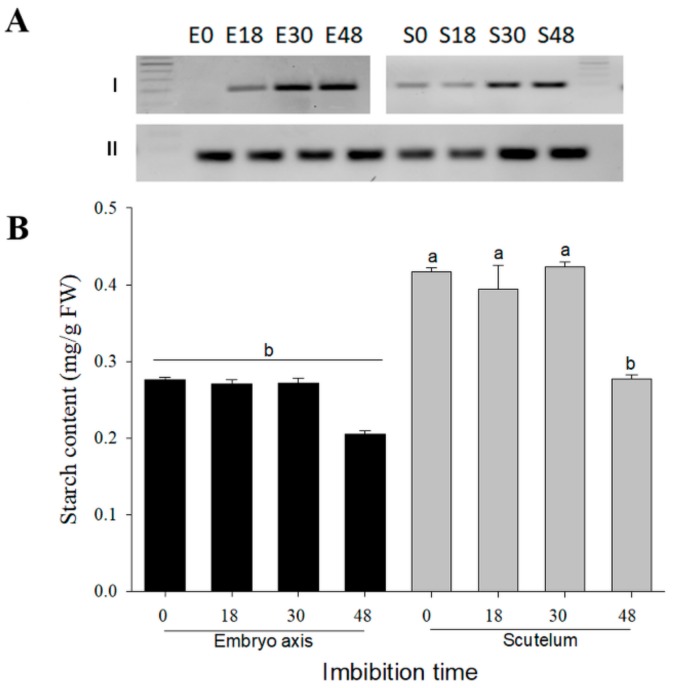
mRNA profile of α-amylase (**A**) and starch content (**B**). (A) Transcripts of I: α-amylase and II: 18s in embryo axis (E) and scutellum (S). (B) Starch content per gram of fresh tissue according to imbibition time. Bars show the average from two independent experiments with triplicates ± SD. Different letters indicate statistically significant values according to the Tukey test, *p* < 0.05.

Starch granules have been detected in embryonic tissues [41] and may contribute to the hexose content in the embryo axis. The starch content and the α-amylase mRNA presence were analyzed during germination to explore this possibility. The starch degradation contributes with sugars after the radicle protrusion since the level of transcripts of the α-amylase is high on phase III of germination (Figure 6A) and the starch hydrolysis begins after 48 h (Figure 6B). Consequently, the increase in the hexose content at the embryo axis at 18 and 30 h of germination suggests a high availability of Suc, which is moved from the scutellum, and active Suc catabolism.

### 3.4. The Sugar Transport along the Germination in the Embryonic Tissues Is Mainly Diffusional

**Figure 7:**
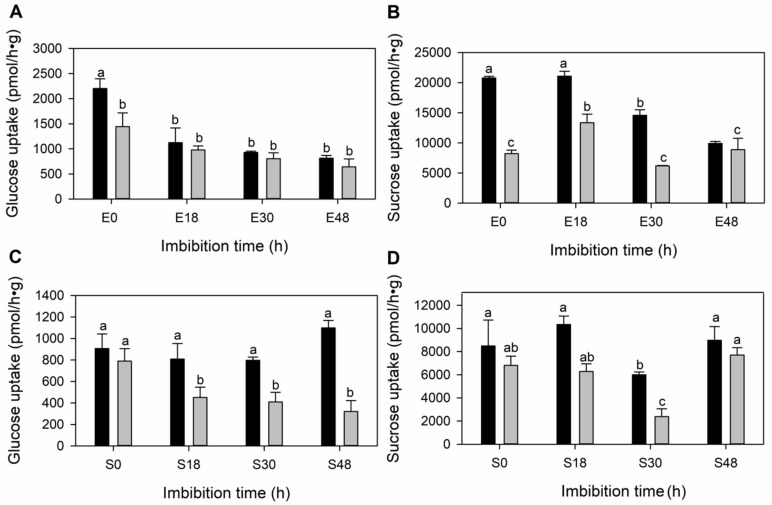
Sugar uptake in embryo axis (E) and scutellum (S) tissue isolated from seeds imbibed at different times. (**A**,**B**) Embryo axis, (**C**,**D**) scutellum, (**A**) and (**C**) ^14^C-glucose uptake, (**B**) and (**D**) ^14^C-sucrose uptake. Dark bars indicate the total sugar uptake, and grey bars show the diffusional sugar uptake average ± SD. Glucose diffusional uptake was determined by adding 10 μM of the protonophore CCCP to the transport medium and the sucrose diffusional uptake was the result of adding a general modifier of sulfhydryl groups, 2.5 mM PCMBS, to the transport medium. Determination was made in triplicates. Different letters indicate statistically significant values according to the Tukey test, *p* < 0.05.

To determine if sugars are moved by active or diffusional transporters, the scutellum and embryo axis were incubated in ^14^C labelled Glc and Suc to detect the total uptake. The contribution of the sugar diffusional uptake to the total transport activity was determined by adding active transporter inhibitors to the medium. The ionophore CCCP inhibits the Glc uptake dependent on a proton gradient. The sulfhydryl modifying reagent PCMBS has been classically used to inhibit transporters; Suc transporters have cysteines that are modified by this compound [32]. We found that the total uptake was partially inhibited, suggesting that both active and diffusional transporters contribute to the total sugar uptake (Figure 7). The total Suc uptake is at least five times higher than the total Glc uptake in both tissues. After adding the active transporter inhibitor the Glc and Suc uptake represents more than 50% of the total uptake activity in both the scutellum and embryo axis, except this uptake occurs at 0 and 18 h in the scutellum and embryo axis and at 30 h in the scutellum; this means that the Glc and Suc diffusional uptake is taking place. The embryo axis (Figure 7A,B) showed a higher sugar uptake than the scutellum (Figure 7C,D). At 18 h of imbibition, the high content of sucrose in the embryo axis (Figure 4A) occurs simultaneously with the highest capacity of Suc diffusional uptake (Figure 7B).

### 3.5. The Abundance of Sugars Affects the Embryo Axis ZmSWEET mRNA Content

**Figure 8:**
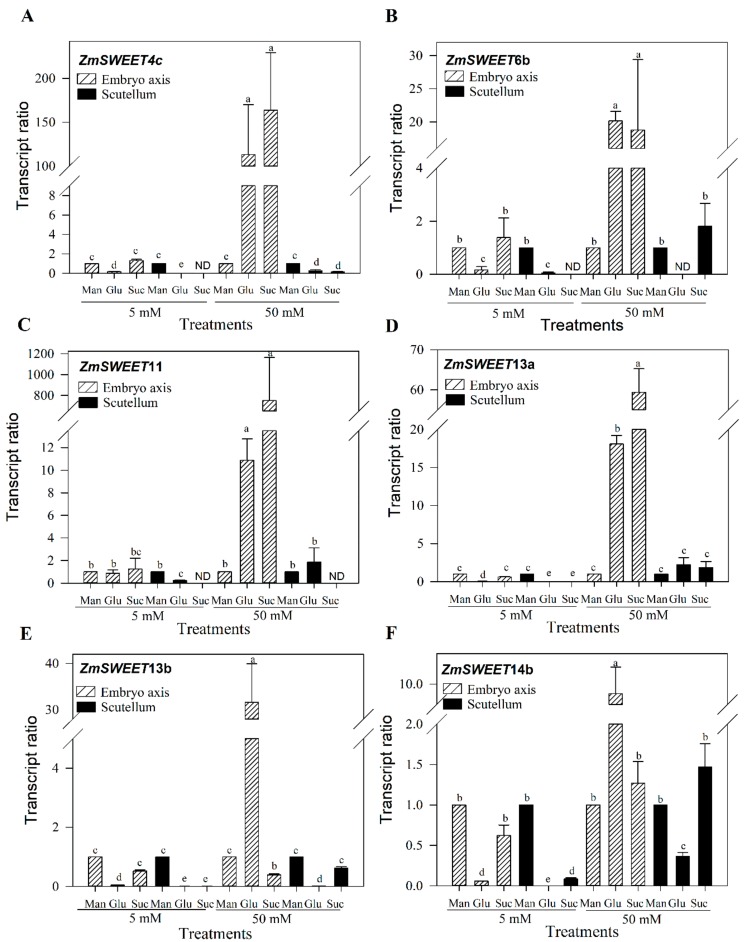
Effect of glucose, sucrose and mannitol in ZmSWEET mRNA levels in the embryo axis and scutellum. (**A**) ZmSWEET4c, (**B**) ZmSWEET6b, (**C**) ZmSWEET11, (**D**) ZmSWEET13a, (**E**) ZmSWEET13b, (**F**) ZmSWEET14b. Bars represent the average of two independent experiments with two technical replicates, each ± SD. Relative transcript amount was calculated according to the Pfaffl method [30], normalizing with the invariant housekeeping gene *18s* and the transcripts of each ZmSWEET from tissues that were obtained from germinated seeds in 5 or 50 mM mannitol. Different letters indicate statistically significant values according to the Tukey test, *p* < 0.05. ND: not detected.

Sugar availability varies according to the germination time and tissue (Figure 4) and could affect the SWEET transcripts amount, a possibility that was suggested to regulate the ZmSWEET4c level of transcripts at embryogenesis [10]. To determine the effect of sugars in the mRNA content of the most abundant SWEETs during germination, seeds were germinated for 24 h in agar supplemented with 5 or 50 mM Glc and Suc; thereafter, the scutellum and embryo axis were isolated. Embryonic tissues obtained from seeds germinated in agar with mannitol were used as the osmotic control. The embryo axis level of SWEET transcripts was the most affected but only when the tissue was incubated with 50 mM of sugar; Glc and Suc induced the increase on mRNAs of all ZmSWEETs analyzed except for ZmSWEET13b and ZmSWEET14b with 50 mM Suc (Figure 8). At the scutellum, 50 mM Glc reduced the mRNA levels of ZmSWEET13b and ZmSWEET14b.

## 4. Discussion

Germination is a key process that allows the seed to develop into a photosynthetic organism. Typically, the germination is described by considering the oxygen and water uptake curves in three phases. In phase I, the water uptake is required for metabolic activity to resume; for example, the respiratory activity increases upon imbibition because of the activation of the mitochondrial enzymes and transport proteins. During phase II, even though low or no water uptake is detected, a further increase in the metabolism takes place in association with the accelerated synthesis of proteins and nucleic acids. Phase III initiates after radicle protrusion, and marks the beginning of the seedling development [11]. To sustain these activities during the first two phases of the germination, the embryonic tissues must mobilize their accumulated metabolites. In phase III, the breakdown of the storage reserves at the endosperm supports the seedling development [11,42]. We studied some of the events that take place in the three phases of germination; in order to do so, the scutellum and the embryo axis were isolated at 0, 18 and 30 h to cover phases I and II of germination, and for phase III the embryonic tissues were isolated at 48 h.

The mobilization process during germination includes the metabolism and transport of molecules. Mono- and disaccharides are hydrophilic solutes that barely diffuse across cell membranes; thus, they must be transported cell-to-cell through plasmodesmata [43] or by specific sugar transporters [44]. Here, we examined the levels of transcripts of diffusional sugar putative transporters, the SWEETs, along the maize embryo germination to show their relevance in the context of the carbon metabolic changes occurring during the germination process. 

We found that the scutellum and the embryo axis isolated from dry and imbibed seeds have the capacity for uptaking Suc and Glc by active and diffusional uptake. Glc transport was detected in rice embryos during germination [18]. A reduction of approximately 60% in Glc transport during germination and seedling development was detected in the *stp1* Arabidopsis mutant. STP1 is a symporter which can transport Glc, D-galactose and D-mannose [45]. In maize, the sucrose uptake and efflux were detected in the scutellum, and the efflux was not related to the proton gradient [46]. Embryos and embryo axis isolated from the maize seeds and later imbibed at different times were able to transport Suc and Glc to the tissues, but the Glc uptake was higher than the sucrose uptake [16]. That result contrasts with the one found in the present work; differences in the varieties of maize could account for the transport activity detected in those lines. However, another explanation is possible; traditionally the study of the events that occur in the different phases of germination was made using isolated embryos from dry seeds which were then germinated. The isolated embryo is able to produce enzymes and molecules that naturally export to the endosperm, such as the gibberellins used to induce the synthesis of α amylase. However, the endosperm needs to be attached to the embryo to induce, for example, the cell wall remodeling enzymes which act in the cells near the radicle. The structural changes occurring in the endosperm, such as cell wall degradation, alters the type and concentration of sugars that may impact the embryo transport capability. In addition, the endosperm content of absicisic acid and gibberellic acid could affect the embryo reserve mobilization and even inhibit the germination [47]. All of these changes may impact in the transport specificity of the scutellum and could explain the differences between the work of Sánchez-Linares et al [16] and the present work. The induction of the SWEET transcription when the seeds were imbibed in 50 mM Suc or Glc supports the idea that changes in the type and temporal mobilization of the reserves impact the sugar transport activity. 

Suc transport activity in the scutellum and embryo axis was expected since it is known that during reserve storage mobilization, the reserves are initially converted into sucrose (Suc) either in the endosperm or the aleurone layer, which can be imported by SUTs [48] or SWEETs into the scutellum. The low CWINV activity in the embryonic tissues may explain the high Suc uptake capacity of the scutellum and embryo axis along the three phases of germination (Figure 7). Therefore, Suc and hexose uptakes by active and diffusional transporters could be taking place at the same time to ensure that all the carbon sources, such as Glc and Suc that come from the adjacent tissues, will be used for the embryo axis as it develops into a new plant. 

Diffusional uptake is possible at the scutellum and the embryo axis since they contain vascular systems in which sugars may move through the symplastic pathway that involves the plasmodesmata; however, such cell-to-cell communication is not evenly distributed in the parenchyma cells or in the embryo axis cells, so a diffusional transporter could be involved [14,20] The concurrence of symplastic and apoplastic pathways to transport sugars has been described in leaves for sugar phloem loading; both transport pathways are important to the plant development [4].

**Figure 9:**
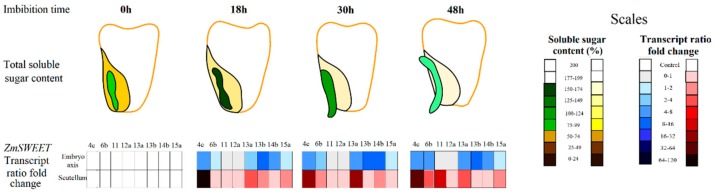
Total soluble sugar content and ZmSWEET transcription level in embryo axis and scutellum along germination. Scales were normalized to total soluble sugar levels with 0 h of imbibition as a control. Transcripts levels determined by qPCR are expressed as a fold of change between 0 h and the indicated times.

The expression of SUTs in the scutellum has been described in wheat, barley, rice and maize [16,19,49,50]. Here we show that not only active transporters are involved in the sugar transport during germination. A different set of SWEET transcripts in maize embryonic tissues are present in the time span studied (Figure 2). All the SWEETs examined have low abundance in dry seed. In the scutellum, transcripts of SWEET4c and SWEET13a are the most abundant (Figure 2C,D). In the embryo axis, transcripts of SWEET4c, SWEET11, SWEET13a, SWEET13b and SWEET14b increase as the germination proceeds (Figure 2A,B). During the embryogenesis, SWEET4c is highly expressed in the basal endosperm layer transference (BELT) in maize, allowing import of the Glc to filial tissues [10]; therefore, the presence of SWEET4c transcripts in the embryonic tissues was expected. However, in the dry seed, no or low mRNA amount of SWEETs was found and an important amount of sugars are present in the embryonic tissues (Figure 9). The increase in ZmSWEET mRNAs coincides with the accumulation of sugars in the embryo axis at 18 h of germination (Figure 9). The ZmSWEET13 family are plasma membrane Suc transporters, and they are important for Suc phloem loading [33], and the presence of transcripts for them in embryonic tissues may explain the detected Suc diffusional uptake. SWEETs are grouped into four clades [35], and the members of each clade seem to share a similar sugar preference [35]; therefore, phylogenetically related transporters are expected to have similar biochemical characteristics. Taking this into consideration, SWEET4b and SWEET6b could be Glc transporters like SWEET4c, and SWEET11, SWEET12a, SWEET14b and SWEET15b could be Suc transporters like members of the ZmSWEET13 family. However, such sugar specificity is yet to be determined.

The scutellum and the endosperm are storage tissues that provide their reserves to the embryo axis, but they are mobilized at different times (Figure 9). The endosperm is rich in starch and this molecule’s breakdown starts late in germination [11,42]. We determined that the scutellum and the embryo axis also have starch (Figure 6), but as reported for the starch in the endosperm [42], this sugar is hydrolyzed during phase III. That could be due to the null or minimal transcription of the α-amylase in the first two phases of the germination and its high level of transcription in phase III. The starch mobilization also coincides with the low sugar content at the scutellum (Figure 9). The α-amylase is de novo synthesized in the embryo and in the aleurone layer together with other enzymes that hydrolyze the starch, and they are exported to the endosperm [42]. The products of the starch hydrolysis include Glc, the disaccharide mannose, oligosaccharides, and Glc-1P [42]. The high abundance of *SWEET4c* and *SWEET13a* mRNA indicates that the scutellum has the capacity to transport Glc and Suc; no information exists on whether SWEETs can transport maltose. However, SUT1a, b and d from *Triticum aestivum* [19] and SUT2 in *A. thaliana* transport mannose with low affinity [51]. Here, we found SUT1 mRNA highly represented during phase III in both scutellum and embryo axis. Possibly, SUT1, together with the SWEETs, are important during seedling development for distribute the massive sugars that are produced from the breakdown of the starch in the endosperm during phase III. In addition, as the SWEETs are bidirectional transporters [44], their presence at the scutellum could help to release Glc and Suc in the apoplast to feed the embryo axis, if the concentration gradient is optimal to drive this movement. Carpaneto et al. [52] suggested that the SUT could drive Suc efflux, and its presence in the scutellum and aleurone layer could indicate that function [16,18,19]; however, the best candidate to fulfill that function is a diffusional transporter, and the SWEETs have been proven to operate as diffusional sugars in several systems and for different plants [4,6,28,29].

The scutellum is considered an absorptive tissue, but it also has stored reserves that are enough to support the embryo axis development into a new plant without the participation of the stored endosperm reserves [11,16,42]. The scutellum has the capacity to synthesize Suc not only from the Glc that comes from the endosperm [19] but also from its lipid reserves through the active lipolysis, activation of the glyoxylate cycle and the Suc synthesis pathway [16]. Suc accumulation could occur since the tissue expresses the main maize Suc symporter, SUT1. 

Despite the active transcription of SUT1, MAS and SPS, the Suc content declines in the scutellum during germination, and one possibility is that Suc is used to sustain its metabolism, but the hexose levels are maintained, which may happen if high phosphorylating activity on the hexoses is reached as proposed by [16,53]. Another possibility is that the Suc is exported for the nourishment of the embryo axis, which may explain why Suc levels in the imbibed embryo axis are high or at the same level as in the dry tissue, and may also justify the increased hexose levels in the embryo axis since the embryo axis does not have Suc biosynthesis capability and does not start the starch degradation until late in the germination process. The protein that facilitates the sugar export from the scutellum has not been described yet, but the presence of SWEET transcripts in the scutellum is relevant because it indicates that the efflux of sugars that has been proposed that occurs for the nourishment of the embryo axis could be carried out by SWEETs. The abundance of SWEET13a, a known Suc transporter, is indicative that it may participate in the efflux of Suc from the scutellum. SWEET14b also increases during germination, and its participation in the sugar transport at the scutellum cannot be discounted.

SWEET13a and SWEET13b transcripts are abundant not only at the scutellum but also at the embryo axis. RNA-Seq analysis showed that the SWEET13 family is highly expressed in maize leaves at later development stages [54]. The abundance of the ZmSWEET13 members suggests a role in the Suc efflux from the scutellum to the embryo axis and in the influx from the apoplast to the embryo axis. The ortholog in rice OsSWEET14 is involved in pathogen susceptibility. *Xanthomonas oryzae* TAL (transcription activator-like) effector AVRXa7 binds to the *SWEET* promotor, inducing its expression so that it might increase the Suc efflux from the infected plant cell [3]. ZmSWEET13a and SWEET13b are interesting targets to study during such maize–pathogen interactions since the seeds usually deal with such stress.

Phylogenetically related transporters, AtSWEET11 and AtSWEET12, have Suc efflux activity and are localized in the plasma membrane of parenchyma veins cells in the leaves [55]. Mutant sweet11:sweet12 plants are chlorotic, grow slowly and accumulate starch in the leaves when growing under high light conditions [55], suggesting that SWEET11 and SWEET12 have an important role in Suc mobilization from source to sink tissues. ZmSWEET11 and ZmSWEET14b also belong to the same clade and their mRNA presence in embryo axis increases during germination.

SWEET4c is not only abundant in the scutellum but also in the embryo axis. Its ortholog in *A. thaliana*, AtSWEET4, is expressed in the primary root stele and cotyledons [56], tissues which come from the embryo axis development. AtSWEET4 is able to transport Glc [3] and Fru [56]. If the ZmSWEET4 family also transports both hexoses, then it could be an advantage for the embryo axis since cell wall invertases produce Glc and Fru from the hydrolysis of Suc. The increase in abundance of ZmSWEET4c transcripts after the incubation with Glc and Suc suggests that the availability of sugars can modulate the sugar partitioning during germination.

In addition, the high mRNA amount of SWEETs (Figure 2) together with the SUT1 (Figure 3) is indicative that both diffusional and active transport are relevant to the embryo axis nourishment during germination.

## 5. Conclusions

We identified transcripts for known transporters—*SWEET*4c, *SWEET*13a and *SWEET*13b—as well as for putative SWEETs, *SWEET*6b, *SWEET*11 and *SWEET*14b, which are abundant during the maize germination process. The level of transcripts was different, depending on the embryonic tissue and imbibition time. This variety of SWEETs may contribute to the high Glc and Suc diffusional uptake detected at the scutellum and embryo axis and suggests that, together with SUT, they have a key role in embryo axis nourishment. In addition, Glc and Suc induction of SWEET mRNA in the embryo axis is indicative that the sugar availability regulates the capability of this tissue to acquire sugars.

## Supplementary Materials

The following are available online at https://www.mdpi.com/2073-4425/10/10/780/s1, Table S1: List of primers used for the transcript analysis by RT-PCR and RT-qPCR. Table S2: Standard curves for qPCR analysis, Table S3: cDNA input for qPCR reaction, Table S4. Cq values of genes analyzed in embryonic tissues (E=Embryo axis, S=scutellum) at different germination times (0, 18, 30 and 48 h). Figure S1: Integrity and purity of RNA and cDNA used for RT-PCR and RT-qPCR analysis. Figure S2: Primer-BLAST analysis. Figure S3: Specific amplification and melt curve obtained from the primers used to RT-qPCR expression analysis. Figure S4: MtN3/saliva domains and seven transmembrane helices predictions of ZmSWEETs. Figure S5: RT-PCR analysis of *ZmSWEETs* mRNA in embryonic tissues along germination.).
