# Supplementary material for: SWEET Transporters for the Nourishment of Embryonic Tissues during Maize Germination
Source: Genes (Basel). 2019 Oct 7;10(10):780. doi: 10.3390/genes10100780 (PMC6826359; doi:10.3390/genes10100780)

**Fig. S1.** Integrity and purity of RNA and cDNA used for RT-PCR and RT-qPCR analysis. A) RNA integrity. Electrophoresis in agarose gel 2%. Bands corresponding to 28s and 18s are shown. No gDNA contamination is visible. B) Representative figure of RNA quantification in Nanodrop 2000 (Thermo Scientific Inc. DE. USA).

**A**)


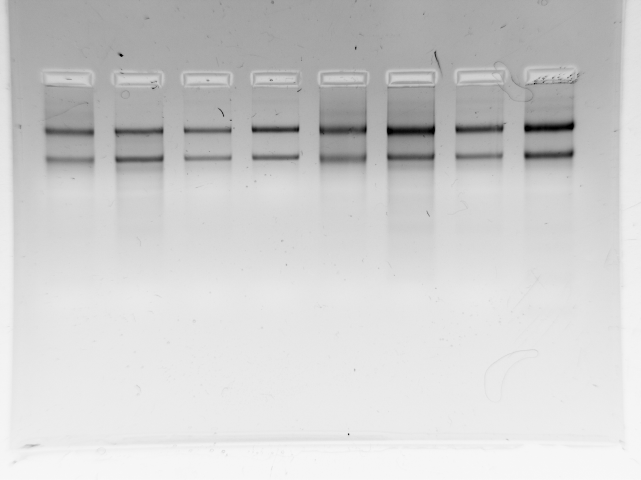


**B)**


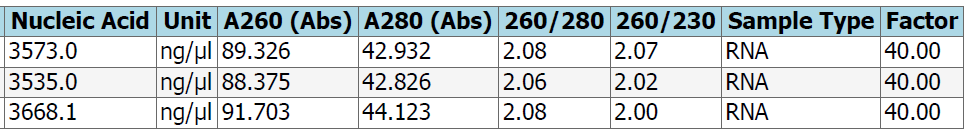

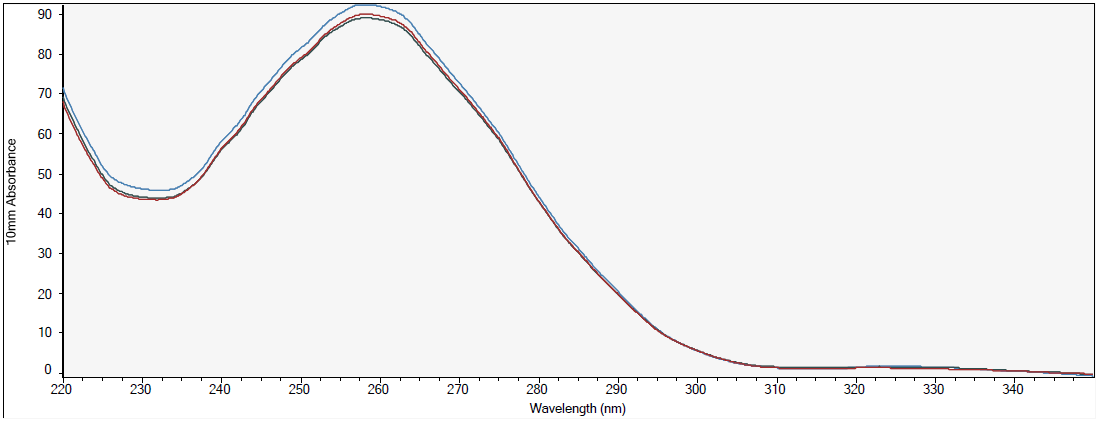

Supplement: Supplementary file 1 [file genes-10-00780-s001.zip › Fig S1.docx]
