# Supplementary material for: SWEET Transporters for the Nourishment of Embryonic Tissues during Maize Germination
Source: Genes (Basel). 2019 Oct 7;10(10):780. doi: 10.3390/genes10100780 (PMC6826359; doi:10.3390/genes10100780)

**Fig. S2.** Primer-BLAST analysis. Exons are show in green boxes and amplification product is show in blue lines. Schemes was obtained using NCBI Nucleotid-BLAST and PRIMER-BLAST tools online.


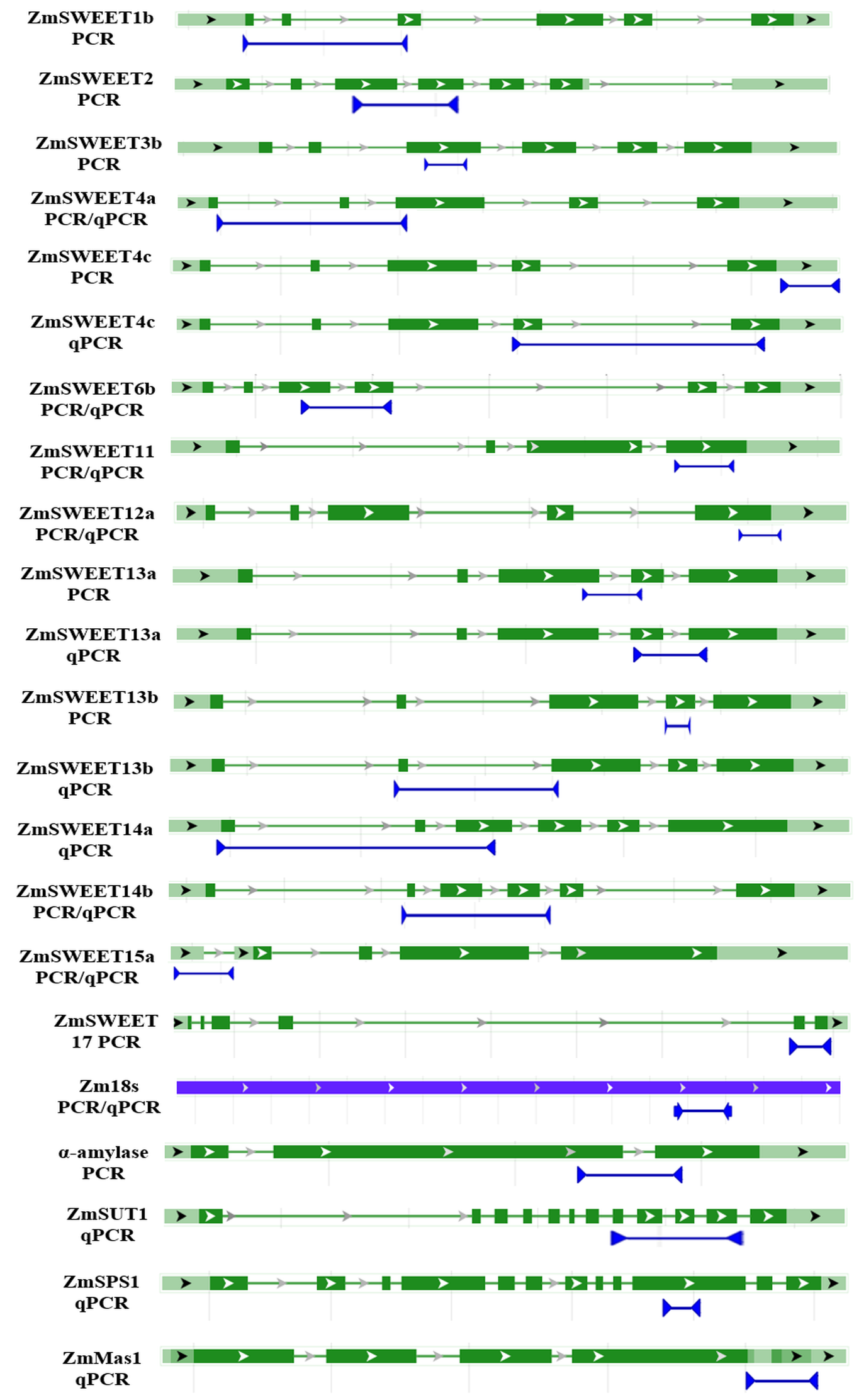

Supplement: Supplementary file 1 [file genes-10-00780-s001.zip › Fig S2.docx]
