# Supplementary material for: SWEET Transporters for the Nourishment of Embryonic Tissues during Maize Germination
Source: Genes (Basel). 2019 Oct 7;10(10):780. doi: 10.3390/genes10100780 (PMC6826359; doi:10.3390/genes10100780)

**Figure S4**. Specific amplification and melt curve from the primers used to RT-qPCR expression analysis.


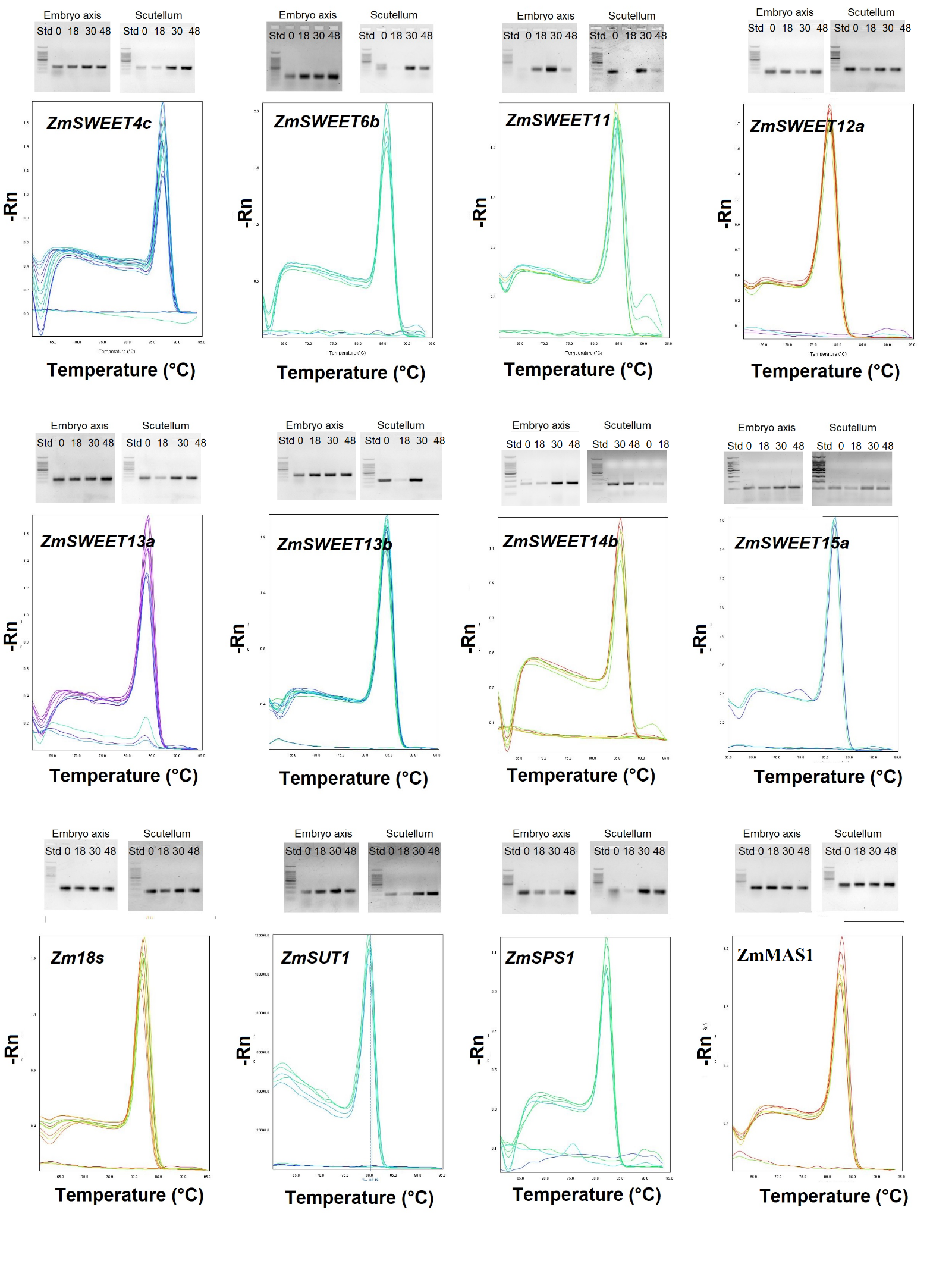

Supplement: Supplementary file 1 [file genes-10-00780-s001.zip › Fig S3.docx]
