# Supplementary material for: SWEET Transporters for the Nourishment of Embryonic Tissues during Maize Germination
Source: Genes (Basel). 2019 Oct 7;10(10):780. doi: 10.3390/genes10100780 (PMC6826359; doi:10.3390/genes10100780)

**Figure S****4**. MtN3/saliva domains and seven transmembrane helices predictions of ZmSWEETs. A) The two MtN3/saliva domains are present in all putative maize SWEET proteins. B) Most of these putative proteins possess seven transmembrane helices. The domain identification was made with Pfam 31.0 database and transmembrane prediction was performed with TMHMM Server version 2.0.

**A)**


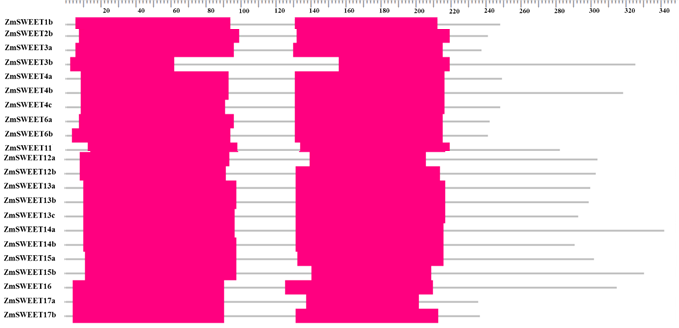


**B)**


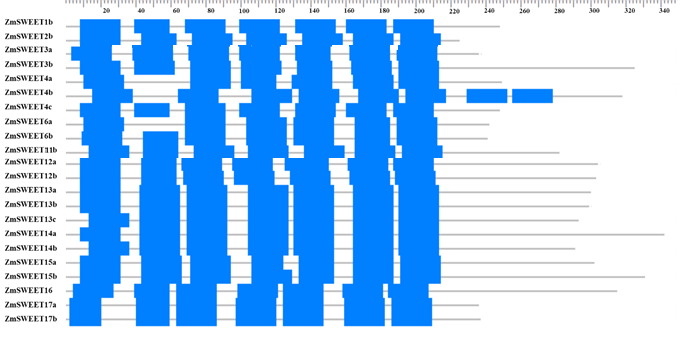

Supplement: Supplementary file 1 [file genes-10-00780-s001.zip › Fig S4.docx]
