# Supplementary material for: SWEET Transporters for the Nourishment of Embryonic Tissues during Maize Germination
Source: Genes (Basel). 2019 Oct 7;10(10):780. doi: 10.3390/genes10100780 (PMC6826359; doi:10.3390/genes10100780)
Supplement: Supplementary file 1 [file genes-10-00780-s001.zip › Fig S5.docx]

**Figure S5.** RT-PCR analysis of *ZmSWEET* mRNAs in embryonic tissues along germination. E, embryo; S, scutellum, at different times of imbibition (0, 18, 30 and 48 hours). A) *ZmSWEET*s that shows increment of their expression along germination. B) *ZmSWEET* without changes in expression along germination. C) *ZmSWEET* expressed only in post-germinative phase. D) Endogenous gene Zm18s expression. The densitometry levels of bands were normalized to E0 or S0 densitometry band respectively. Bars represent the meaning ±SD of three independent biological samples. Different letters indicate statistically significant values according to the Tukey test, p <0.05. Representative agarose-gel images are shown.

**A)**


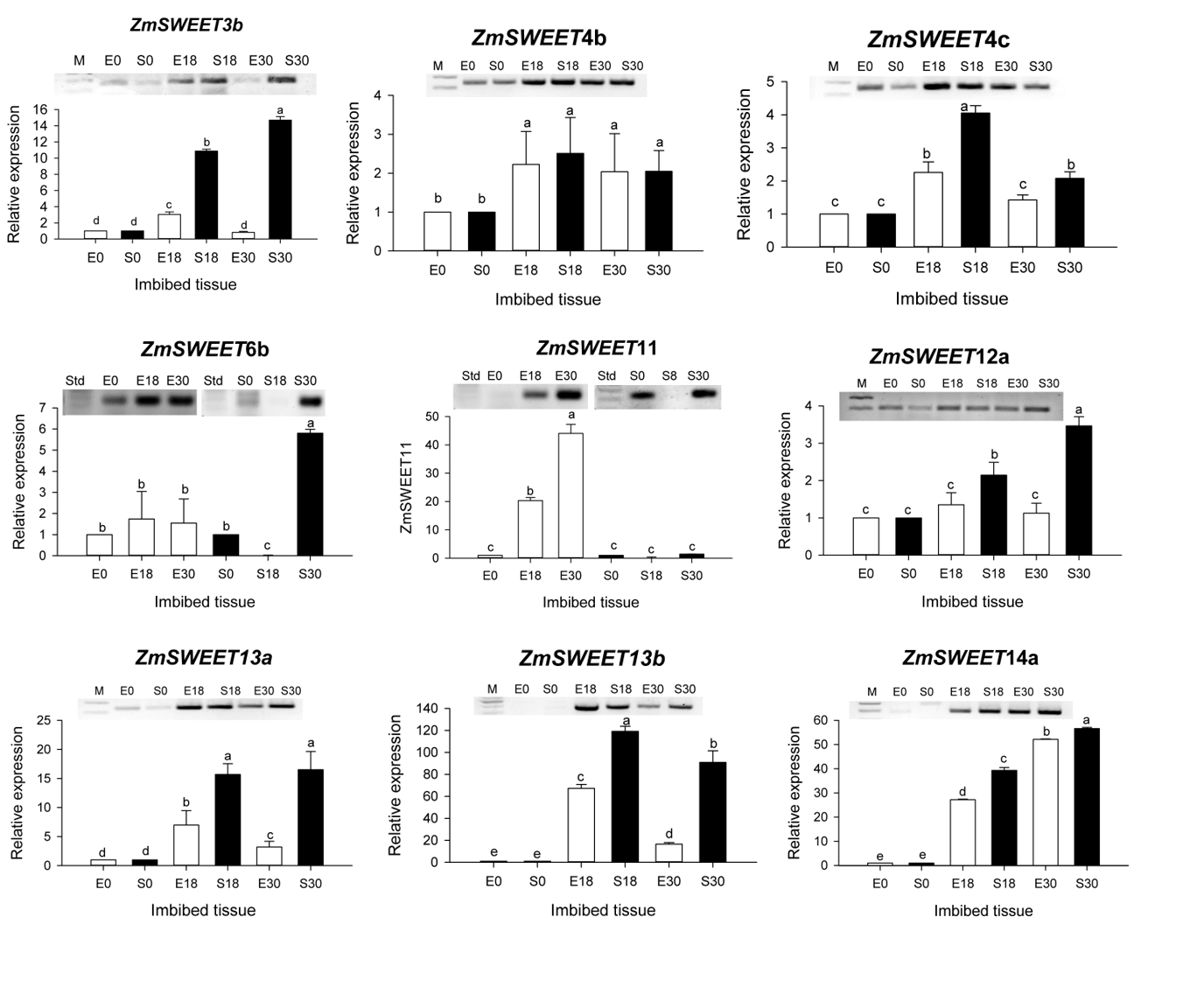


**B)**


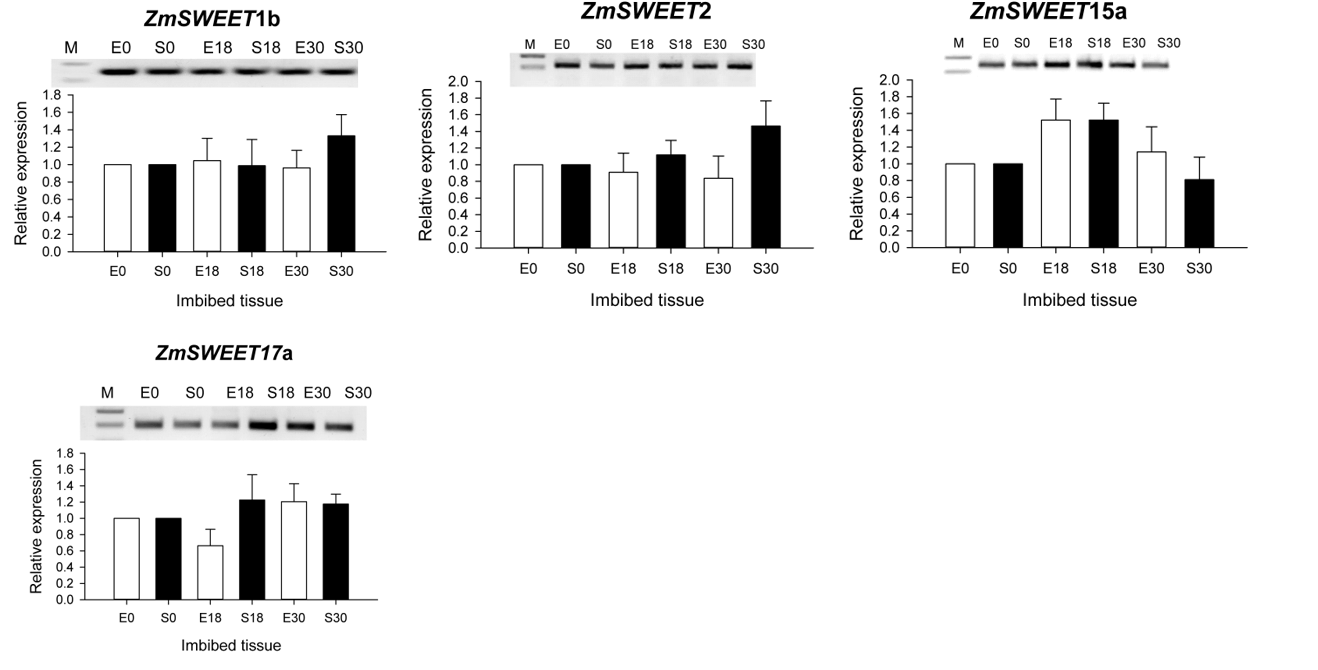


**C)**


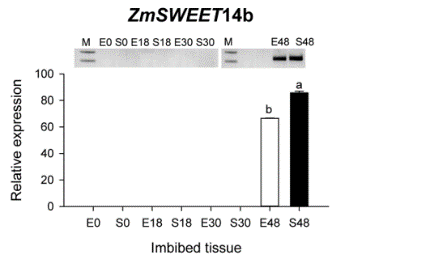


**D)**
