# Supplementary material for: SWEET Transporters for the Nourishment of Embryonic Tissues during Maize Germination
Source: Genes (Basel). 2019 Oct 7;10(10):780. doi: 10.3390/genes10100780 (PMC6826359; doi:10.3390/genes10100780)
Supplement: Supplementary file 1 [file genes-10-00780-s001.zip › Table S1.docx]

**Table S1**. List of primers used for the transcript analysis by RT-PCR and RT-qPCR.

| **Gene** | **Aplication** | **Primers pair** | **Size product** | **Description** | **Max Score** | **Total Score** | **Query Cover** | **E value** | **Per. Ident** | **NCBI Accesion** |
| --- | --- | --- | --- | --- | --- | --- | --- | --- | --- | --- |
| *ZmSWEET*1b  GRMZM2G153358 | RT-PCR | F 5’-ACCATATAAGCGCAAGCAGACA  R 5’-CAGAACGTAGGCACTGGGG | 151 bp | [Zea mays uncharacterized LOC100281296 (LOC100281296), mRNA](https://blast.ncbi.nlm.nih.gov/Blast.cgi#alnHdr_1511289982) gene_synonym="GRMZM2G153358" | 41 | 76.5 | 85% | 0.003 | 100% | [NM_001367193.1](https://www.ncbi.nlm.nih.gov/nucleotide/NM_001367193.1?report=genbank&log$=nucltop&blast_rank=1&RID=P8CZZMNK015) |
| ZmSWEET2  GRMZM2G324903 | RT-PCR | F 5’- AGGCTCAAGGTCTCTGCTCT  R 5’- TGACGATTGACATGGGGGAC | 163 bp | [Zea mays uncharacterized LOC100279636 (LOC100279636), mRNA](https://blast.ncbi.nlm.nih.gov/Blast.cgi#alnHdr_226508059) gene_synonym="GRMZM2G324903" | 37.4 | 74.7 | 80% | 0.039 | 100% | [NM_001152631.1](https://www.ncbi.nlm.nih.gov/nucleotide/NM_001152631.1?report=genbank&log$=nucltop&blast_rank=5&RID=P8ETTEWV01R) |
| ZmSWEET3b  GRMZM2G060974 | RT-PCR | F 5’- CAGGAGAATCCCTTGTGCGT  R 5’- AACAGTTGGGTCGCTGCTAT | 158 bp | [PREDICTED: Zea mays bidirectional sugar transporter SWEET3b (LOC103649564), mRNA](https://blast.ncbi.nlm.nih.gov/Blast.cgi#alnHdr_1315003852)  gene_synonym=" GRMZM2G060974" | 37.4 | 67.5 | 72% | 0.066 | 100% | [XM_008675300.3](https://www.ncbi.nlm.nih.gov/nucleotide/XM_008675300.3?report=genbank&log$=nucltop&blast_rank=1&RID=P8EVWR1001R) |
| ZmSWEET4c  GRMZM2G137954 | RT-PCR | F 5’-CAGCAAGCAAAAGCCACATA  R 5’-AGCAACAAGACAGCAGCAGA | 218 bp | [Zea mays uncharacterized LOC100273706 (LOC100273706), mRNA](https://blast.ncbi.nlm.nih.gov/Blast.cgi#alnHdr_825706150)  gene_synonym="GRMZM2G137954” | 37.5 | 74.7 | 80% | 0.039 | 100% | [NM_001148118.2](https://www.ncbi.nlm.nih.gov/nucleotide/NM_001148118.2?report=genbank&log$=nucltop&blast_rank=1&RID=P8F95USJ015) |
| ZmSWEET4c GRMZM2G137954 | RT-qPCR | F 5’-CCAGACAAAGAGCGTGGAAT  R 5’-CATCATGGTTGACTGCTGGT | 234 bp | [Zea mays uncharacterized LOC100273706 (LOC100273706), mRNA](https://blast.ncbi.nlm.nih.gov/Blast.cgi#alnHdr_825706150) gene_synonym="GRMZM2G137954” | 37.4 | 74.7 | 80% | 0.039 | 100% | [NM_001148118.2](https://www.ncbi.nlm.nih.gov/nucleotide/NM_001148118.2?report=genbank&log$=nucltop&blast_rank=1&RID=PAN56HXP015) |
| ZmSWEET6b  GRMZM2G416965 | RT-PCR y  RT-qPCR | F 5’-GTCGTCCACCCTAACAGCAT  R 5’-ATCATCGAGCCGAAAATGAC | 244 bp | [Zea mays seven-transmembrane-domain protein 1 (LOC100284352), mRNA](https://blast.ncbi.nlm.nih.gov/Blast.cgi#alnHdr_226532939)  gene_synonym=" GRMZM2G416965” | 37.4 | 70.2 | 80% | 0.039 | 100% | [NM_001157247.1](https://www.ncbi.nlm.nih.gov/nucleotide/NM_001157247.1?report=genbank&log$=nucltop&blast_rank=2&RID=PANVTE0J014) |
| ZmSWEET11  GRMZM2G368827 | RT-PCR y  RT-qPCR | F 5’-AGGCATCGTCGTCTGCTAGT  R 5’-GTTGACACGGCGTACGTAGA | 221bp | [Zea mays uncharacterized LOC100282584 (LOC100282584), mRNA](https://blast.ncbi.nlm.nih.gov/Blast.cgi#alnHdr_1486857867)  gene_synonym="GRMZM2G368827” | 37.4 | 74.7 | 85% | 0.034 | 100% | NM_001155492.2 |
| ZmSWEET12a  GRMZM2G133322 | RT-PCR y  RT-qPCR | F 5’-GACCTGACTATGATTGCTGC  R 5’-GCGTCTACTTGTACCGTGGT | 114 bp | [Zea mays uncharacterized LOC100217087 (LOC100217087), mRNA](https://blast.ncbi.nlm.nih.gov/Blast.cgi#alnHdr_219362526)  gene_synonym="GRMZM2G133322" | 37.4 | 74.7 | 80% | 0.040 | 100% | [NM_001143456.1](https://www.ncbi.nlm.nih.gov/nucleotide/NM_001143456.1?report=genbank&log$=nucltop&blast_rank=1&RID=P8M902CW01R) |
| ZmSWEET13a  GRMZM2G173669 | RT-PCR | F 5’-CGAGACCATCTACGTCGTCA  R 5’-CGTCTGGATCACTTCGTCTTCA | 232 bp | [Zea mays SWEET13a (LOC100282708), mRNA](https://blast.ncbi.nlm.nih.gov/Blast.cgi#alnHdr_1590643909)  gene_synonym=" GRMZM2G173669" | 37.4 | 64.8 | 79% | 0.038 | 100% | [NM_001155615.2](https://www.ncbi.nlm.nih.gov/nucleotide/NM_001155615.2?report=genbank&log$=nucltop&blast_rank=1&RID=P8MAH36P01R) |
| ZmSWEET13a  GRMZM2G173669 | RT-qPCR | F 5’-CGTGGAGTACATGCCCTTCT  R 5’-CACGTAGAGCACCATCTGGA | 150 bp | [Zea mays SWEET13a (LOC100282708), mRNA](https://blast.ncbi.nlm.nih.gov/Blast.cgi#alnHdr_1590643909)  gene_synonym=" GRMZM2G173669" | 37.4 | 74.4 | 80% | 0.039 | 100% | [NM_001155615.2](https://www.ncbi.nlm.nih.gov/nucleotide/NM_001155615.2?report=genbank&log$=nucltop&blast_rank=1&RID=PAMJJ1XJ015) |
| ZmSWEET13b GRMZM2G021706 | RT-PCR | F 5’-ACAAATACGTCGCGCGCTACCA  R 5’-GCTTGCTTGCGATGATGGAG | 361 bp | [Zea mays SWEET13b (LOC100273779), mRNA](https://blast.ncbi.nlm.nih.gov/Blast.cgi#alnHdr_226532045)  gene_synonym=" GRMZM2G021706" | 37.4 | 66.6 | 80% | 0.043 | 100% | [NM_001148182.1](https://www.ncbi.nlm.nih.gov/nucleotide/NM_001148182.1?report=genbank&log$=nucltop&blast_rank=1&RID=P8N2JTZF01R) |
| ZmSWEET13b  GRMZM2G021706 | RT-qPCR | F 5’-GACCTTCCTAGCCCCGATAC  R 5’-GAACAGCTTGGCTTTCTTGG | 254 bp | [Zea mays SWEET13b (LOC100273779), mRNA](https://blast.ncbi.nlm.nih.gov/Blast.cgi#alnHdr_226532045)  gene_synonym=" GRMZM2G021706" | 37.4 | 70.2 | 80% | 0.040 | 100% | [NM_001148182.1](https://www.ncbi.nlm.nih.gov/nucleotide/NM_001148182.1?report=genbank&log$=nucltop&blast_rank=3&RID=PAMM8X6A015) |
| ZmSWEET14a GRMZM2G094955 | RT-PCR | F 5’-AAATGGCTGGCCTGTCTCTA  R 5’-TGATGAGCAGGAACTCGTTG | 240 bp | [Zea mays sugars will eventually be exported transporter14a (LOC100194326), mRNA](https://blast.ncbi.nlm.nih.gov/Blast.cgi#alnHdr_212723299)  gene_synonym=" GRMZM2G094955" | 37.4 | 74.7 | 80% | 0.039 | 100% | [NM_001139364.1](https://www.ncbi.nlm.nih.gov/nucleotide/NM_001139364.1?report=genbank&log$=nucltop&blast_rank=1&RID=P8F8AVXX015) |
| ZmSWEET14b GRMZM2G015976 | RT-PCR y  RT-qPCR | F 5’-GTCATCGAGACCCTCTAC  R 5’-ACGAAGACGCTAACGGAGAA | 200 bp | [Zea mays mtN3-like protein (LOC100192602), mRNA](https://blast.ncbi.nlm.nih.gov/Blast.cgi#alnHdr_212722953) gene_synonym="GRMZM2G015976" | 37.4 | 71.1 | 79% | 0.036 | 100% | [NM_001137817.1](https://www.ncbi.nlm.nih.gov/nucleotide/NM_001137817.1?report=genbank&log$=nucltop&blast_rank=1&RID=P8N3ZP7801R) |
| ZmSWEET15a  GRMZM2G168365 | RT-PCR y  RT-qPCR | F 5’-CCCTGGCCTCTTCTTCGTTC  R 5’-CCTCGCTTACAGCCCTTCTC | 107 bp | [Zea mays uncharacterized LOC100282648 (LOC100282648), mRNA](https://blast.ncbi.nlm.nih.gov/Blast.cgi#alnHdr_1708352854)  gene_synonym=" GRMZM2G168365" | 37.4 | 74.7 | 80% | 0.039 | 100% | [NM_001371874.1](https://www.ncbi.nlm.nih.gov/nucleotide/NM_001371874.1?report=genbank&log$=nucltop&blast_rank=1&RID=P8N6FAZB01R) |
| ZmSWEET17a  GRMZM2G106462 | RT-PCR | F 5’-TCATGCCGTTCTTCCTATCC  R 5’-GAGGCGACGCTATTTCTTTG | 195 bp | [PREDICTED: Zea mays bidirectional sugar transporter SWEET17 (LOC103650117), mRNA](https://blast.ncbi.nlm.nih.gov/Blast.cgi#alnHdr_1315004171)  gene_synonym=" GRMZM2G106462" | 37.4 | 74.7 | 80% | 0.041 | 100% | XM_008675772.3 |
| Zm18S ribosomal RNA  XR_002748750.1 | RT-PCR y  RT-qPCR | F 5’-CCATCCCTCCGTAGTTAGCTTCT  R 5’-CCTGTCGGCCAAGGCTATATAC | 152 bp | [PREDICTED: Zea mays Eukaryotic 18S ribosomal RNA (LOC111589664),](https://blast.ncbi.nlm.nih.gov/Blast.cgi#alnHdr_1315014396) | 42.8 | 83.7 | 81% | 0.001 | 100% | [XR_002748750.1](https://www.ncbi.nlm.nih.gov/nucleotide/XR_002748750.1?report=genbank&log$=nucltop&blast_rank=1&RID=P8RT4WBN014) |
| Zmα-amylase  GRMZM2G138468 | RT-PCR | F 5’- GTCACGTTCGTCGACAAC  R 5’-TTGACCTCACCGCAGACA | 270 bp | [Zea mays alpha-amylase (LOC542522), mRNA](https://blast.ncbi.nlm.nih.gov/Blast.cgi#alnHdr_1197977595) | 33.7 | 67.5 | 78% | 0.41 | 100% | [NM_001112069.2](https://www.ncbi.nlm.nih.gov/nucleotide/NM_001112069.2?report=genbank&log$=nucltop&blast_rank=1&RID=P8UK9YW7014) |
| ZmSUT1 GRMZM2G034302 | RT-qPCR | F 5’-TCCTCTGGCTCCACAAACAAC  R 5’-ACGAGCTGAATCCTAGAACGA | 463 bp | [Zea mays sucrose transporter 1 (LOC541615), transcript variant 1, mRNA](https://blast.ncbi.nlm.nih.gov/Blast.cgi#alnHdr_809281411) | 39.2 | 73.8 | 80% | 0.012 | 100% | [NM_001111370.3](https://www.ncbi.nlm.nih.gov/nucleotide/NM_001111370.3?report=genbank&log$=nucltop&blast_rank=1&RID=P8TWTMXK01R) |
| ZmSPS1  GRMZM5G875238 | RT-qPCR | F 5’-TGAGAAGAGCTCGCTGAACA  R 5’-GCAGTCCACAGCTATGACGA | 202 bp | [Zea mays sucrose phosphate synthase 1 (LOC542711), mRNA](https://blast.ncbi.nlm.nih.gov/Blast.cgi#alnHdr_1206271560) | 37.4 | 74.7 | 80% | 0.039 | 100% | [NM_001112224.2](https://www.ncbi.nlm.nih.gov/nucleotide/NM_001112224.2?report=genbank&log$=nucltop&blast_rank=2&RID=P8TZCF51015) |
| ZmMas1 GRMZM2G102183 | RT-qPCR | F 5’-CACCGTGCAAGCTCTGAATA  R 5’-CCCGAGAGTTCAACAGAAGC | 236 bp | [PREDICTED: Zea mays malate synthase 1 (mas1), transcript variant X1, mRNA](https://blast.ncbi.nlm.nih.gov/Blast.cgi#alnHdr_1315000332) | 37.4 | 37.4 | 40% | 0.039 | 100% | [XM_008670416.3](https://www.ncbi.nlm.nih.gov/nucleotide/XM_008670416.3?report=genbank&log$=nucltop&blast_rank=1&RID=P8UAARCV014) |
