# Supplementary material for: SWEET Transporters for the Nourishment of Embryonic Tissues during Maize Germination
Source: Genes (Basel). 2019 Oct 7;10(10):780. doi: 10.3390/genes10100780 (PMC6826359; doi:10.3390/genes10100780)
Supplement: Supplementary file 1 [file genes-10-00780-s001.zip › Table S2.docx]

**Table S2**. Standard curves for qPCR analysis.

| **Gene target** | **Slope** | **Y-inter** | **R^2^** | **Efficiencies (E=10^-1/m^)** |
| --- | --- | --- | --- | --- |
| *Zm18s* | -2.895 | 11.384 | 0.993 | 2.215252 |
| *ZmSWEET4c* | -2.988 | 25.795 | 0.985 | 2.161086 |
| *ZmSWEET6b* | -2.923 | 21.756 | 0.908 | 2.198438 |
| *ZmSWEET11* | -4.78 | 23.09 | 0.983 | 1.618844 |
| *ZmSWEET 12a* | -3.053 | 23.63 | 0.989 | 2.125919 |
| *ZmSWEET 13a* | -2.82 | 23.01 | 0.937 | 2.262611 |
| *ZmSWEET 13b* | -3.78 | 21.297 | 0.997 | 1.838867 |
| *ZmSWEET 14b* | -2.957 | 24.611 | 0.957 | 2.178615 |
| *ZmSWEET 15a* | -2.957 | 26.655 | 0.987 | 2.178615 |
| *ZmSUT1* | -3.297 | 15.852 | 0.956 | 2.010509 |
| *ZmSPS1* | -3.146 | 15.225 | 0.993 | 2.079045 |
| *ZmMAS1* | -3.489 | 18.299 | 0.999 | 1.934706 |
