# Supplementary material for: SWEET Transporters for the Nourishment of Embryonic Tissues during Maize Germination
Source: Genes (Basel). 2019 Oct 7;10(10):780. doi: 10.3390/genes10100780 (PMC6826359; doi:10.3390/genes10100780)
Supplement: Supplementary file 1 [file genes-10-00780-s001.zip › Table S3.docx]

**Table S3**. cDNA input for qPCR reaction.

| **Tissue** | **cDNA input of biological sample 1 (ng/µL)** | **cDNA input of biological sample 2 (ng/µL)** |
| --- | --- | --- |
| Embryo axis 0h (E0) | 1115.7 | 989.4 |
| Embryo axis 18h (E18) | 1092.6 | 1003.5 |
| Embryo axis 30h (E30) | 1117.1 | 1287.9 |
| Embryo axis 48h (E48) | 1128.3 | 1165.6 |
| Scutellum 0h (S0) | 1180.3 | 1109.9 |
| Scutellum 18h (S18) | 1195.1 | 1173.4 |
| Scutellum 30h (S30) | 1105.1 | 1255.8 |
| Scutellum 45h (S48) | 1179.6 | 1162.1 |
