# Supplementary material for: SWEET Transporters for the Nourishment of Embryonic Tissues during Maize Germination
Source: Genes (Basel). 2019 Oct 7;10(10):780. doi: 10.3390/genes10100780 (PMC6826359; doi:10.3390/genes10100780)
Supplement: Supplementary file 1 [file genes-10-00780-s001.zip › Table S4.docx]

**Table S4.** Cq values of genes analyzed in embryonic tissues (E=Embryo axis, S=scutellum) at different germination times (0, 18, 30 and 48 h). Cq values of no-RT control and difference between both conditions (Cq noRT- Cq RT) also are show.

|  | **Cq no-RT control** | | | | | | **Cq RT** | | | | | |  |
| --- | --- | --- | --- | --- | --- | --- | --- | --- | --- | --- | --- | --- | --- |
|  | ***Zm18s*** | | | | | | | | | | | | |
| **Tissue** | **Biological sample 1** | | **Biological sample 2** | | **Mean** | **Cv** | **Biological sample 1** | | **Biological sample 2** | | **Mean** | **Cv** | **∆Cq (no RT-RT)** |
| E0 | 21.884 | 21.886 | 20.562 | 20.377 | 21.177 | 0.821 | 13.478 | 13.624 | 14.458 | 13.324 | 13.721 | 0.506 | 7.456 |
| E18 | 21.546 | 21.413 | 20.824 | 20.768 | 21.138 | 0.399 | 13.180 | 14.839 | 12.709 | 13.554 | 13.571 | 0.914 | 7.567 |
| E30 | 23.545 | 23.289 | 22.681 | 21.510 | 22.756 | 0.906 | 14.242 | 14.521 | 14.369 | 14.032 | 14.291 | 0.207 | 8.465 |
| E48 | 19.658 | 19.532 | 21.430 | 21.337 | 20.489 | 1.035 | 13.234 | 14.517 | 13.404 | 13.956 | 13.778 | 0.581 | 6.711 |
| S0 | 21.023 | 21.074 | 20.475 | 20.282 | 20.713 | 0.395 | 12.562 | 12.525 | 12.588 | 12.995 | 12.668 | 0.220 | 8.046 |
| S18 | 20.602 | 20.364 | 19.701 | 19.628 | 20.074 | 0.483 | 12.021 | 12.054 | 11.971 | 11.876 | 11.981 | 0.078 | 8.093 |
| S30 | 22.690 | 22.554 | 23.293 | 23.142 | 22.920 | 0.354 | 14.614 | 14.569 | 14.608 | 13.998 | 14.447 | 0.300 | 8.473 |
| S48 | 20.098 | 19.890 | 19.429 | 19.350 | 19.692 | 0.361 | 14.273 | 14.266 | 14.443 | 14.565 | 14.387 | 0.144 | 5.305 |
| ***ZmSWEET4c*** | | | | | | | | | | | | | |
| **Tissue** | **Biological sample 1** | | **Biological sample 2** | | **Mean** | **Cv** | **Biological sample 1** | | **Biological sample 2** | | **Mean** | **Cv** | **∆Cq (no RT-RT)** |
| E0 | 38.945 | 38.990 | 39.358 | 39.221 | 39.129 | 0.195 | 32.169 | 32.374 | 32.756 | 32.922 | 32.555 | 0.345 | 6.573 |
| E18 | 35.318 | 35.294 | 33.123 | 35.223 | 34.739 | 1.078 | 26.181 | 26.048 | 27.259 | 26.898 | 26.597 | 0.578 | 8.143 |
| E30 | 29.641 | 29.475 | 31.054 | 31.679 | 30.462 | 1.077 | 23.195 | 22.858 | 24.788 | 24.772 | 23.903 | 1.022 | 6.559 |
| E48 | 38.342 | 38.454 | 38.916 | 38.049 | 38.440 | 0.360 | 24.032 | 24.228 | 24.862 | 25.109 | 24.558 | 0.510 | 13.883 |
| S0 | 39.635 | 38.330 | 39.704 | 39.035 | 39.176 | 0.639 | 37.313 | 37.121 | 26.214 | 37.133 | 34.445 | 5.488 | 4.731 |
| S18 | 35.325 | 37.629 | 34.149 | 34.827 | 35.482 | 1.510 | 22.283 | 22.425 | 28.569 | 27.437 | 25.179 | 3.295 | 10.304 |
| S30 | 34.000 | 32.575 | 34.176 | 34.266 | 33.754 | 0.794 | 24.441 | 25.274 | 20.859 | 23.520 | 23.524 | 1.915 | 10.231 |
| S48 | 31.856 | 32.183 | 32.008 | 31.493 | 31.885 | 0.293 | 17.277 | 18.392 | 20.298 | 18.663 | 18.658 | 1.247 | 13.227 |
| ***ZmSWEET6b*** | | | | | | | | | | | | | |
| **Tissue** | **Biological sample 1** | | **Biological sample 2** | | **Mean** | **Cv** | **Biological sample 1** | | **Biological sample 2** | | **Mean** | **Cv** | **∆Cq (no RT-RT)** |
| E0 | 33.378 | 39.222 | 38.317 | 36.074 | 36.748 | 2.607 | 31.383 | 31.612 | 31.823 | 31.602 | 31.605 | 0.180 | 5.143 |
| E18 | 38.897 | 38.300 | 38.458 | 38.699 | 38.589 | 0.263 | 30.524 | 30.633 | 30.739 | ND | 30.632 | 0.108 | 7.957 |
| E30 | 32.242 | 32.135 | 38.143 | 38.701 | 35.305 | 3.606 | 29.918 | 30.076 | 30.227 | 30.071 | 30.073 | 0.126 | 5.232 |
| E48 | 39.839 | 34.969 | 38.495 | 38.487 | 37.947 | 2.085 | 29.476 | 29.171 | 28.850 | 29.156 | 29.163 | 0.256 | 8.784 |
| S0 | 36.387 | 35.319 | 34.821 | 32.802 | 34.832 | 1.503 | 0.000 | 0.000 | 0.000 | 0.000 | 0.000 | 0.000 | 34.832 |
| S18 | 35.489 | 33.497 | 28.176 | 28.586 | 31.437 | 3.625 | ND | ND | ND | ND | 0.000 | 0.000 | 31.437 |
| S30 | 32.650 | 31.080 | 33.768 | 32.649 | 32.537 | 1.105 | ND | ND | ND | ND | 0.000 | 0.000 | 32.537 |
| S48 | 38.265 | 38.790 | 38.615 | 39.583 | 38.813 | 0.558 | 28.462 | 28.607 | 28.741 | ND | 28.603 | 0.140 | 10.210 |
| ***ZmSWEET11a*** | | | | | | | | | | | | | |
| **Tissue** | **Biological sample 1** | | **Biological sample 2** | | **Mean** | **Cv** | **Biological sample 1** | | **Biological sample 2** | | **Mean** | **Cv** | **∆Cq (no RT-RT)** |
| E0 | 39.499 | 38.949 | 39.857 | 39.024 | 39.332 | 0.426 | 32.654 | 33.151 | 35.978 | 34.278 | 34.015 | 1.474 | 5.317 |
| E18 | 34.608 | 36.454 | 35.228 | 34.460 | 35.187 | 0.907 | 27.812 | 27.732 | 28.769 | 28.746 | 28.265 | 0.570 | 6.923 |
| E30 | 38.562 | 38.239 | 37.998 | 37.221 | 38.005 | 0.572 | 23.234 | 23.451 | 23.007 | 22.677 | 23.092 | 0.331 | 14.913 |
| E48 | 39.632 | 38.453 | 39.405 | 38.102 | 38.898 | 0.737 | 22.879 | 21.648 | 21.872 | 21.784 | 22.046 | 0.563 | 16.852 |
| S0 | 35.193 | 34.767 | 35.208 | 35.130 | 35.075 | 0.208 | ND | ND | ND | ND | ND | ND | ND |
| S18 | 39.174 | 39.400 | 39.302 | 39.146 | 39.255 | 0.118 | 37.421 | 37.299 | 35.836 | 38.973 | 37.382 | 1.282 | 1.873 |
| S30 | 39.522 | 39.463 | 39.834 | 39.866 | 39.672 | 0.208 | 32.751 | 34.187 | 30.564 | 30.458 | 31.990 | 1.806 | 7.682 |
| S48 | 39.814 | 39.567 | 39.329 | 38.954 | 39.416 | 0.366 | 31.597 | 31.287 | 35.283 | 37.067 | 33.809 | 2.831 | 5.607 |
| ***ZmSWEET12a*** | | | | | | | | | | | | | |
| **Tissue** | **Biological sample 1** | | **Biological Sample 2** | | **Mean** | **Cv** | **Biological sample 1** | | **Biological sample 2** | | **Mean** | **Cv** | **∆Cq (no RT-RT)** |
| E0 | 35.121 | 35.344 | 35.103 | 35.037 | 35.151 | 0.133 | 20.428 | 21.039 | 20.925 | 20.906 | 20.824 | 0.271 | 14.327 |
| E18 | 33.986 | 34.001 | 33.472 | 34.384 | 33.961 | 0.374 | 23.241 | 24.085 | 24.457 | 24.438 | 24.055 | 0.569 | 9.906 |
| E30 | 35.463 | 35.215 | 35.211 | 34.757 | 35.162 | 0.294 | 24.574 | 25.854 | 23.440 | 23.825 | 24.423 | 1.064 | 10.738 |
| E48 | 35.053 | 35.419 | 35.149 | 34.804 | 35.106 | 0.254 | 27.479 | 27.802 | 27.778 | 28.147 | 27.802 | 0.273 | 7.305 |
| S0 | 34.202 | 32.688 | 34.321 | 34.344 | 33.889 | 0.803 | 22.634 | 22.730 | 23.576 | 23.378 | 23.080 | 0.468 | 10.809 |
| S18 | 32.425 | 35.701 | 35.238 | 33.601 | 34.241 | 1.509 | 26.377 | 26.460 | 31.072 | 31.769 | 28.919 | 2.902 | 5.322 |
| S30 | 34.296 | 34.871 | 35.998 | 35.883 | 35.262 | 0.819 | 27.985 | 28.204 | 27.584 | 27.822 | 27.899 | 0.262 | 7.363 |
| S48 | 35.919 | 36.167 | 34.984 | 35.634 | 35.676 | 0.510 | 28.121 | 28.779 | 29.971 | 28.961 | 28.958 | 0.766 | 6.718 |
| ***ZmSWEET13a*** | | | | | | | | | | | | | |
| **Tissue** | **Biological sample 1** | | **Biological Sample 2** | | **Mean** | **Cv** | **Biological sample 1** | | **Biological sample 2** | | **Mean** | **Cv** | **∆Cq (no RT-RT)** |
| E0 | 34.662 | 32.192 | 38.868 | 38.956 | 36.170 | 3.324 | 28.770 | 28.830 | 28.480 | 28.630 | 28.678 | 0.156 | 7.492 |
| E18 | 38.828 | 28.907 | 27.259 | 26.827 | 30.455 | 5.653 | 23.600 | 23.540 | 23.660 | 23.830 | 23.658 | 0.125 | 6.798 |
| E30 | 39.172 | 35.142 | 37.087 | 29.631 | 35.258 | 4.096 | 20.490 | 20.900 | 21.590 | 21.630 | 21.153 | 0.554 | 14.105 |
| E48 | 39.699 | 39.604 | 39.090 | 38.183 | 39.144 | 0.694 | 19.850 | 19.960 | 19.840 | 19.940 | 19.898 | 0.061 | 19.246 |
| S0 | 37.545 | 37.958 | 37.631 | 37.903 | 37.759 | 0.202 | 30.510 | 34.990 | 31.990 | 31.520 | 32.253 | 1.927 | 5.507 |
| S18 | 38.752 | 38.261 | 36.696 | 36.223 | 37.483 | 1.214 | 27.415 | 28.530 | 24.930 | 28.880 | 27.439 | 1.785 | 10.044 |
| S30 | 32.105 | 32.943 | 32.260 | 32.322 | 32.408 | 0.368 | 22.530 | 24.020 | 20.150 | 20.110 | 21.703 | 1.915 | 10.705 |
| S48 | 30.762 | 35.230 | 35.753 | 32.001 | 33.436 | 2.436 | 19.060 | 18.970 | 27.600 | 28.140 | 23.443 | 5.117 | 9.994 |
| ***ZmSWEET13b*** | | | | | | | | | | | | | |
| **Tissue** | **Biological sample 1** | | **Biological Sample 2** | | **Mean** | **Cv** | **Biological sample 1** | | **Biological sample 2** | | **Mean** | **Cv** | **∆Cq (no RT-RT)** |
| E0 | 38.692 | 37.586 | 38.435 | 38.104 | 38.204 | 0.477 | 30.826 | 31.515 | 31.776 | 31.187 | 31.326 | 0.412 | 6.878 |
| E18 | 28.421 | 29.387 | 28.844 | 27.610 | 28.566 | 0.750 | 19.243 | 18.722 | 22.630 | 22.683 | 20.820 | 2.132 | 7.746 |
| E30 | 36.167 | 32.800 | 30.451 | 30.978 | 32.599 | 2.583 | 19.132 | 18.961 | 20.692 | 20.781 | 19.891 | 0.979 | 12.708 |
| E48 | 39.763 | 36.978 | 32.453 | 37.947 | 36.785 | 3.111 | 18.965 | 17.821 | 17.960 | 17.823 | 18.142 | 0.552 | 18.643 |
| S0 | 39.343 | 32.975 | 34.925 | 35.977 | 35.805 | 2.666 | 21.692 | 22.206 | 22.294 | 22.445 | 22.159 | 0.327 | 13.646 |
| S18 | 30.199 | 35.267 | 28.598 | 28.799 | 30.715 | 3.117 | 21.879 | 21.301 | 26.351 | 25.308 | 23.710 | 2.496 | 7.006 |
| S30 | 39.357 | 37.263 | 37.117 | 38.547 | 38.071 | 1.071 | 25.133 | 24.848 | 19.236 | 19.369 | 22.146 | 3.286 | 15.925 |
| S48 | 37.968 | 37.481 | 37.190 | 34.946 | 36.896 | 1.339 | 17.669 | 17.828 | 19.915 | 21.917 | 19.332 | 2.004 | 17.564 |
| ***ZmSWEET14b*** | | | | | | | | | | | | | |
| **Tissue** | **Biological sample 1** | | **Biological Sample 2** | | **Mean** | **Cv** | **Biological sample 1** | | **Biological sample 2** | | **Mean** | **Cv** | **∆Cq (no RT-RT)** |
| E0 | 39.091 | 39.487 | 39.763 | 39.039 | 39.345 | 0.343 | 37.031 | 36.221 | 36.373 | 35.284 | 36.227 | 0.720 | 3.118 |
| E18 | 35.639 | 34.597 | 35.675 | 37.546 | 35.864 | 1.228 | 25.231 | 27.805 | 26.510 | 26.305 | 26.463 | 1.056 | 9.402 |
| E30 | 38.545 | 38.443 | 37.902 | 34.989 | 37.470 | 1.678 | 24.768 | 26.458 | 25.758 | 25.847 | 25.708 | 0.700 | 11.762 |
| E48 | 36.836 | 36.095 | 36.066 | 35.273 | 36.068 | 0.638 | 34.838 | 35.015 | 25.759 | 26.040 | 30.413 | 5.214 | 5.655 |
| S0 | 36.642 | 36.954 | 38.687 | 38.866 | 37.787 | 1.152 | 26.261 | 27.002 | 27.471 | 27.928 | 27.165 | 0.711 | 10.622 |
| S18 | 36.519 | 35.355 | 38.121 | 35.488 | 36.371 | 1.277 | 27.931 | 27.912 | 28.076 | 27.771 | 27.923 | 0.125 | 8.448 |
| S30 | 34.563 | 34.102 | 33.993 | 34.042 | 34.175 | 0.262 | 24.600 | 24.441 | 24.600 | 24.145 | 24.446 | 0.215 | 9.729 |
| S48 | 34.795 | 34.748 | 32.976 | 36.841 | 34.840 | 1.580 | 25.406 | 25.075 | 33.400 | 33.092 | 29.243 | 4.626 | 5.597 |
| ***ZmSWEET15a*** | | | | | | | | | | | | | |
| **Tissue** | **Biological sample 1** | | **Biological Sample 2** | | **Mean** | **Cv** | **Biological sample 1** | | **Biological sample 2** | | **Mean** | **Cv** | **∆Cq (no RT-RT)** |
| E0 | 39.211 | 39.455 | 39.159 | 39.129 | 39.238 | 0.148 | 30.518 | 32.402 | 31.157 | 31.051 | 31.282 | 0.797 | 7.957 |
| E18 | 36.364 | 34.606 | 31.829 | 32.236 | 33.759 | 2.125 | 24.795 | 24.892 | 26.106 | 25.850 | 25.411 | 0.664 | 8.348 |
| E30 | 35.414 | 36.674 | 36.620 | 32.301 | 35.252 | 2.052 | 24.715 | 24.846 | 25.930 | 25.688 | 25.295 | 0.605 | 9.957 |
| E48 | 34.993 | 39.223 | 39.515 | 36.206 | 37.484 | 2.235 | 29.712 | 28.439 | 25.428 | 26.023 | 27.401 | 2.017 | 10.084 |
| S0 | 32.357 | 31.884 | 32.638 | 34.421 | 32.825 | 1.109 | 25.119 | 25.702 | 24.984 | 25.550 | 25.339 | 0.342 | 7.486 |
| S18 | 33.771 | 32.831 | 32.734 | 32.849 | 33.046 | 0.485 | 23.654 | 24.004 | 25.975 | 27.184 | 25.204 | 1.669 | 7.842 |
| S30 | 38.606 | 39.642 | 39.635 | 37.907 | 38.947 | 0.847 | 32.489 | 34.247 | 25.058 | 25.803 | 29.399 | 4.648 | 9.548 |
| S48 | 37.233 | 37.151 | 32.961 | 35.531 | 35.719 | 1.998 | 24.048 | 23.895 | 24.784 | 25.126 | 24.463 | 0.588 | 11.256 |
| ***ZmSUT1*** | | | | | | | | | | | | | |
| **Tissue** | **Biological sample 1** | | **Biological Sample 2** | | **Mean** | **Cv** | **Biological sample 1** | | **Biological sample 2** | | **Mean** | **Cv** | **∆Cq (no RT-RT)** |
| E0 | 38.864 | 39.198 | 38.698 | 38.584 | 38.836 | 0.267 | 28.978 | 27.766 | 27.745 | 27.642 | 28.033 | 0.633 | 10.803 |
| E18 | 28.584 | 28.765 | 28.917 | 28.200 | 28.616 | 0.309 | 20.905 | 20.829 | 21.653 | 21.829 | 21.304 | 0.511 | 7.313 |
| E30 | 29.220 | 29.406 | 29.010 | 28.803 | 29.110 | 0.261 | 17.319 | 17.067 | 17.643 | 17.403 | 17.358 | 0.238 | 11.752 |
| E48 | 27.899 | 28.318 | 28.204 | 28.871 | 28.323 | 0.406 | 16.581 | 16.317 | 16.494 | 16.378 | 16.442 | 0.118 | 11.880 |
| S0 | 39.148 | 39.606 | 39.007 | 39.708 | 39.368 | 0.342 | 33.640 | 33.730 | 36.702 | 34.975 | 34.762 | 1.430 | 4.606 |
| S18 | 36.757 | 36.400 | 38.479 | 38.302 | 37.485 | 1.059 | 31.311 | 30.675 | 23.912 | 23.162 | 27.265 | 4.323 | 10.219 |
| S30 | 31.412 | 31.467 | 31.738 | 33.534 | 32.038 | 1.008 | 29.334 | 28.512 | 16.472 | 16.963 | 22.820 | 7.058 | 9.217 |
| S48 | 28.672 | 28.262 | 28.922 | 28.798 | 28.664 | 0.286 | 14.334 | 14.775 | 24.872 | 25.750 | 19.933 | 6.223 | 8.731 |
| ***ZmSPS1*** | | | | | | | | | | | | | |
| **Tissue** | **Biological sample 1** | | **Biological Sample 2** | | **Mean** | **Cv** | **Biological sample 1** | | **Biological sample 2** | | **Mean** | **Cv** | **∆Cq (no RT-RT)** |
| E0 | 37.985 | 37.054 | 37.964 | 37.062 | 37.516 | 0.529 | 26.766 | 26.848 | 29.108 | 29.230 | 27.988 | 1.365 | 9.528 |
| E18 | 35.341 | 36.868 | 35.889 | 37.820 | 36.479 | 1.094 | 30.778 | 30.510 | 31.983 | 30.924 | 31.049 | 0.646 | 5.431 |
| E30 | 33.651 | 34.333 | 32.290 | 34.474 | 33.687 | 0.998 | 28.471 | 28.370 | 28.760 | 28.587 | 28.547 | 0.168 | 5.140 |
| E48 | 34.197 | 33.136 | 35.413 | 33.460 | 34.051 | 1.010 | 27.798 | 28.130 | 29.365 | 29.227 | 28.630 | 0.783 | 5.421 |
| S0 | 39.448 | 39.568 | 36.621 | 39.557 | 38.798 | 1.453 | 30.635 | 31.981 | 31.310 | 31.304 | 31.307 | 0.549 | 7.491 |
| S18 | 39.346 | 39.406 | 38.921 | 39.815 | 39.372 | 0.366 | 34.186 | 34.307 | 34.246 | 34.247 | 34.247 | 0.049 | 5.126 |
| S30 | 35.869 | 35.865 | 36.841 | 35.612 | 36.047 | 0.543 | 39.711 | 30.886 | 26.392 | 26.553 | 30.885 | 6.241 | 5.161 |
| S48 | 34.830 | 34.910 | 34.693 | 34.665 | 34.775 | 0.115 | 26.370 | 26.248 | 31.394 | 30.879 | 28.723 | 2.795 | 6.052 |
| ***ZmMAS1*** | | | | | | | | | | | | | |
| **Tissue** | **Biological sample 1** | | **Biological Sample 2** | | **Mean** | **Cv** | **Biological sample 1** | | **Biological sample 2** | | **Mean** | **Cv** | **∆Cq (no RT-RT)** |
| E0 | 37.840 | 37.456 | 38.596 | 37.534 | 37.856 | 0.520 | 20.438 | 20.444 | 20.364 | 20.623 | 20.467 | 0.110 | 17.389 |
| E18 | 38.067 | 35.631 | 38.247 | 38.466 | 37.603 | 1.325 | 18.923 | 19.051 | 19.091 | 19.019 | 19.021 | 0.072 | 18.582 |
| E30 | 36.625 | 36.435 | 34.753 | 36.199 | 36.003 | 0.851 | 16.828 | 16.920 | 17.263 | 17.061 | 17.018 | 0.190 | 18.985 |
| E48 | 38.121 | 37.421 | 38.326 | 37.312 | 37.795 | 0.504 | 17.767 | 17.832 | 17.990 | 18.268 | 17.964 | 0.223 | 19.831 |
| S0 | 37.515 | 37.786 | 36.928 | 37.027 | 37.314 | 0.406 | 26.355 | 26.106 | 20.495 | 21.206 | 23.541 | 3.121 | 13.773 |
| S18 | 37.711 | 36.910 | 37.956 | 37.775 | 37.588 | 0.464 | 28.653 | 28.575 | 36.711 | 26.691 | 30.158 | 4.462 | 7.430 |
| S30 | 32.564 | 34.648 | 35.790 | 32.886 | 33.972 | 1.519 | 13.930 | 14.007 | 13.867 | 13.849 | 13.913 | 0.071 | 20.059 |
| S48 | 39.291 | 38.726 | 38.147 | 38.051 | 38.554 | 0.575 | 14.984 | 15.017 | 18.740 | 18.739 | 16.870 | 2.159 | 21.684 |
